# Integrative Genomics Reveals Causal Pleiotropy and Therapeutic Opportunities at the Interface of Acute Pancreatitis and Infection

**DOI:** 10.1002/jhbp.70041

**Published:** 2025-11-27

**Authors:** Bo Zou, Hao Jiang, Jingsheng Ruan, Daifeng Yang, Xing Chen, Shanshan Cai, Xinglin Yi

**Affiliations:** ^1^ Department of Critical Care Medicine Affiliated Hospital of Jiangnan University Wuxi China; ^2^ Department of Otorhinolaryngology–Head and Neck Surgery The First Affiliated Hospital of Naval Medical University Shanghai China; ^3^ Department of Thoracic Surgery Jinshan Hospital of Fudan University Shanghai China; ^4^ School of Computing, Engineering & Digital Technologies Teesside University Middlesbrough UK; ^5^ Department of Gastroenterology First Affiliated Hospital of Army Medical University Chongqing China; ^6^ Division of Biomedical and Life Sciences, Faculty of Health and Medicine Lancaster University Lancaster UK; ^7^ Department of Respiratory and Critical Care Medicine First Affiliated Hospital of Army Medical University Chongqing China

**Keywords:** acute pancreatitis, drug repurposing, genetic pleiotropy, GWAS, infection

## Abstract

**Background:**

Understanding the genetic links between acute pancreatitis (AP) and its infectious comorbidities is crucial for prognosis and therapy, yet remains underexplored.

**Methods:**

We conducted a comprehensive post‐GWAS analysis using large‐scale summary statistics for AP and 16 infectious diseases. To pinpoint pleiotropic genes, we integrated multi‐omics data via transcriptome‐wide and proteome‐wide association studies, and resolved cell‐type‐specific effects using single‐cell analysis. Extensive locus colocalization analyses were performed to validate our findings by estimating the probability of shared causal variants.

**Results:**

This computational discovery phase prioritized 29 high‐confidence pleiotropic genes, including established loci (*SPINK1, CRP*) and novel candidates (*ERBB2, ALDH2, FLOT1*). To functionally validate and contextualize these findings, we performed bulk transcriptomic analysis on peripheral blood from AP patients and employed gsMap, a spatial GWAS mapping algorithm, to integrate our genetic data with transcriptomics from a murine AP model, comparing pathological versus normal tissue. These analyses confirmed that the identified genes are dynamically regulated in a severity‐dependent manner in patients and are activated within specific pathological niches in pancreatic tissue.

**Conclusion:**

In conclusion, this study provides a genetic map linking AP and its infectious comorbidities, offering insights into potential prevention strategies and highlighting novel therapeutic targets for further investigation and validation.

## 1 Introduction

Acute pancreatitis (AP) represents a major gastrointestinal emergency characterized by acute pancreatic parenchymal inflammation, resulting in substantial morbidity and mortality. Global epidemiological data from 1990 to 2021 indicate that AP affects 814 500 individuals aged 15–39 years annually, causing 16 800 deaths in this demographic [1]. The pathophysiology of AP involves the premature activation of digestive enzymes within pancreatic acinar cells, triggering glandular autodigestion and a potent pro‐inflammatory cascade. In severe cases, this localized process can escalate to a systemic inflammatory response syndrome (SIRS) and multi‐organ dysfunction syndrome (MODS) [2]. A pivotal determinant of prognosis in severe AP is the onset of secondary infection. Pancreatic necrosis, a complication in 10%–20% of AP cases, creates an environment ripe for microbial invasion. Translocation of gut bacteria is the primary mechanism leading to infected necrotizing pancreatitis (INP), a condition that develops in a third of patients with necrosis and is associated with mortality rates as high as 40% [2]. The transition from sterile necrosis to infected necrotizing pancreatitis represents a critical inflection point where host defense mechanisms become overwhelmed. The recent COVID‐19 pandemic, which saw an increased prevalence and severity of AP, has further highlighted a potential bidirectional relationship between systemic infections and pancreatic injury [3]. Therefore, elucidating the shared biological pathways that govern both susceptibility to infection and the severity of AP is essential for developing novel therapies to mitigate this devastating complication.

Despite compelling clinical links, the shared genetic architecture between AP and infectious diseases remains largely unexplored. While genome‐wide association studies (GWAS) have identified loci for AP and various infections independently, this single‐trait approach cannot systematically detect pleiotropy—where single genetic variants influence multiple distinct phenotypes. Uncovering these pleiotropic effects is fundamental to understanding the shared heritability and biological mechanisms underlying this clinical comorbidity. Moving from statistical association to biological insight requires sophisticated post‐GWAS analytical frameworks that can interrogate genetic signals across multiple biological layers, from the transcriptome and proteome to specific cellular contexts.

**FIGURE 1:**
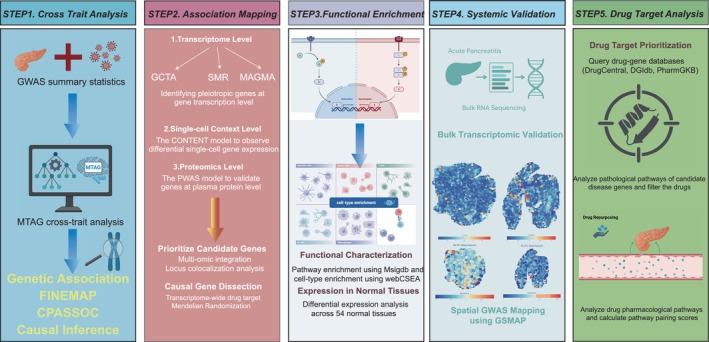
Graphical abstract of the study design.

Here, we present a comprehensive post‐GWAS investigation to dissect the shared genetic etiology between acute pancreatitis and a diverse panel of infectious diseases, including sepsis, streptococcal septicaemia, and tuberculosis, among others. First, we aimed to identify the fundamental genetic architecture and pleiotropic loci that confer a shared susceptibility to both systemic inflammation and a broad spectrum of infections. As outlined in our study design (Figure 1), this is achieved through a multi‐layered analytical strategy that employs cross‐trait analyses, transcriptome‐wide (TWAS), and proteome‐wide (PWAS) association studies to infer causality. We systematically examined the genetic relationships between AP and 16 infectious conditions, including sepsis, streptococcal septicemia, intestinal infectious diseases (IID), viral hepatitis (VH), acute lower respiratory infection (ALRI), skin and subcutaneous infections (SSI), cystitis, and acute tubulointerstitial nephritis (ATIN), among others. Second, we aim to functionally validate these genetic findings and translate them into a pathophysiological context. To achieve this, we leverage bulk transcriptomic data from human AP patients to confirm the clinical relevance of the identified genes, specifically by examining their expression levels in patients with severe AP, the subgroup most vulnerable to infection. Furthermore, we employ advanced spatial transcriptomic analysis in a murine AP model to pinpoint the specific cellular microenvironments within the pancreas where these genetic pathways are activated during the disease process.

## 2 Methods

### 2.1 Data Acquisition and Quality Control

GWAS summary statistics for AP and 16 distinct infectious conditions were obtained from publicly available datasets, primarily sourced from the FinnGen consortium (R12) and Verma's GWAS statistics [4]. The infectious diseases investigated were sepsis, streptococcal septicemia, *Clostridium difficile* enterocolitis, candidiasis, IID, bacterial infections, mycoses, VH, infectious mononucleosis, ALRI, central nervous system inflammatory conditions, tuberculosis, herpes zoster, cytomegalovirus infection, skin and SSI, cystitis, and ATIN (Table S1). This comprehensive selection of infectious phenotypes was designed to identify core, pleiotropic pathways of host defense, rather than to model only specific clinical sequelae of AP.

To ensure the validity and robustness of the genetic data for downstream analyses, we performed a rigorous quality control process. We utilized linkage disequilibrium score regression (LDSC) to estimate the single‐nucleotide polymorphism (SNP)‐based heritability for AP and each infectious disease. The statistical significance of the heritability estimates was evaluated using *Z*‐scores, with a threshold of *Z* > 3 considered indicative of a reliable genetic component suitable for further investigation. Furthermore, the LDSC intercept was examined to assess for potential confounding from factors such as population stratification or cryptic relatedness. Intercepts approximating 1.0 were deemed acceptable, suggesting minimal inflation of test statistics.

### 2.2 Assessment of Genetic Correlation and Causal Inference

We systematically quantified the shared genetic architecture between AP and each of the 16 infectious outcomes using LDSC. This method estimates the genetic correlation between two traits by regressing the product of their GWAS *Z*‐scores against a linkage disequilibrium score. The formula for genetic correlation is: $r_{g}=\mathrm{co}v_{g}/\sqrt{\left(h_{g1}^{2}*h_{g2}^{2}\right)}$, where $\mathrm{co}v_{g}$ represents the genetic covariance between the two traits, and $h_{g1}^{2}$ and $h_{g2}^{2}$ are their respective SNP‐based heritabilities. To account for multiple testing across the 16 trait pairs, *p* values for genetic correlations were adjusted using the Benjamini‐Hochberg procedure to control the false discovery rate (FDR).

To formally evaluate the causal relationships between AP and each infectious disease, we conducted bi‐directional, two‐sample Mendelian Randomization (MR). For each analysis, genetic variants robustly associated with the exposure trait (*p* < 5 × 10^−8^) were selected as potential instrumental variables (IVs). These IVs were subsequently pruned to ensure independence (*r* ^2^ < 0.001 within a 10 000 kb window). To mitigate bias, we filtered out variants with low minor allele frequency (MAF < 0.01) and excluded weak instruments, defined as those with an F‐statistic below 10. The primary causal estimate was derived using the random‐effects inverse‐variance weighted (IVW) model. In cases where an exposure was instrumented by only a single valid IV, the Wald ratio method was employed. A suite of sensitivity analyses was performed to assess the validity of the MR assumptions, including MR‐Egger regression to detect directional pleiotropy and Cochran's Q statistic to evaluate heterogeneity among the IVs. Statistical significance for the causal estimates was determined using FDR correction to account for all bi‐directional tests.

### 2.3 Cross‐Trait Analysis and Identification of Pleiotropic Variants

To enhance statistical power for discovering loci with shared effects between AP and each infectious disease, we implemented two complementary analytical methods. First, Multi‐Trait Analysis of GWAS (MTAG) was applied to each trait pair that demonstrated a significant genetic correlation. MTAG leverages genetic correlation to combine information across traits, generating more precise SNP‐level association estimates and meta‐analysis *p*‐values (*p*_MTAG_). As a complementary approach, we employed CPASSOC to identify pleiotropic variants, a method that accounts for heterogeneity in genetic effects across phenotypes via the SHet test statistic. Variants meeting our predefined significance criteria were then functionally annotated using ANNOVAR, a positional mapping method, to map them to their nearest genes and genomic features.

Subsequently, each significant pleiotropic locus underwent statistical fine‐mapping to identify putative causal variants. This process involved analyzing a ±500 kb genomic region surrounding these SNPs using a Bayesian framework to calculate a posterior inclusion probability (PIP) for each variant. Finally, a 99% credible set was constructed for each locus by ranking variants in descending order of their PIPs and including those with the highest probabilities until the cumulative sum reached or exceeded 0.99.

### 2.4 Multi‐Omic Identification of Pleiotropic Genes

Recognizing that simple positional mapping is often insufficient to identify the effector genes underlying pleiotropic signals, we implemented a comprehensive multi‐omic strategy to infer shared genetic regulators. This involved a suite of six complementary analyses leveraging eQTL data and mixed‐model frameworks. Gene‐level associations were first imputed by aggregating SNP *p*‐values within a gene into a test statistic, with local linkage disequilibrium structure accounted for by MAGMA. These signals were then re‐assessed through a complementary burden test implemented in GCTA‐fastBAT, which operates on a mixed‐model approximation from summary statistics. To investigate the role of genetically regulated gene expression, we then conducted several TWAS analyses. These included using sparse‐Canonical Correlation Analysis (sCCA) with the Aggregated Cauchy Association Test (ACAT) to build robust cross‐tissue expression models, and performing Summary‐data‐based Mendelian Randomization (SMR) to test for causal links between gene expression and disease risk using cis‐eQTL data from relevant tissues (Whole blood and Pancreas) in the GTEx v8 database [5]. Moving to the proteomic level, we conducted a PWAS using pre‐computed prediction models for 4657 plasma proteins to assess the impact of genetically determined protein abundance. Finally, to explore cell‐type‐specific effects, we applied CONtexT‐spEcific‐geNeTics pipeline (CONTENT), a novel method that enhances the power to detect context‐dependent genetic effects by decomposing gene expression into shared and specific components [6]. For all six methodologies, *p*‐values were adjusted for multiple testing using the FDR, with a significance threshold of *p*_FDR_ < 0.05. The European panel from the 1000 Genomes Project (Phase 3) served as the linkage disequilibrium reference for all analyses.

### 2.5 Bayesian Colocalization and Causal Gene Inference

To test whether shared association signals at a pleiotropic locus were driven by a common causal variant, we first performed Bayesian colocalization analysis for each AP‐infection trait pair. For each locus containing a gene implicated by our multi‐omic analyses, we calculated the posterior probability for Hypothesis 4 (PPH4), which represents the probability of a single shared causal variant. A PPH4 threshold greater than 0.6 was considered strong evidence of colocalization.

To further probe the causality of genes at these colocalized loci, we performed a locus‐specific two‐sample MR analysis. For each candidate gene, we selected cis‐acting SNPs from the AP GWAS to serve as IVs. These potential IVs were required to have a *p*‐value < 5 × 10^−3^ and were clumped to ensure independence (clumping window = 250 kb, *r* ^2^ threshold < 0.2). The causal effect of AP on the corresponding infectious disease, mediated by the genetic variants at that specific locus, was then estimated. For loci with multiple independent IVs, the random‐effects IVW method was applied. In cases where only a single valid IV was available, the Wald ratio method was employed.

### 2.6 Functional Annotation and Pathway Analysis

The functional significance of the identified pleiotropic genes was investigated through systematic enrichment analyses against canonical pathways and Gene Ontology (GO) databases. We used the Molecular Signatures Database (MsigDB) to identify overrepresented canonical pathways and gene ontologies. To pinpoint relevant cellular contexts, we conducted cell‐type‐specific enrichment analysis using WebCSEA, which compares the gene list against expression signatures from over 110 distinct tissue–cell types. The FUMA platform was further utilized to characterize the expression patterns of these genes across normal human tissues. All enrichment analyses were corrected for multiple testing using the FDR method.

### 2.7 Bulk Transcriptomic Validation in Human Acute Pancreatitis

To validate the clinical relevance of our identified pleiotropic genes, we performed a differential expression analysis using a publicly available human bulk RNA‐sequencing dataset [7]. This dataset comprises peripheral blood samples from AP patients, categorized as Mild (*n* = 79) and Severe (*n* = 8), as well as from 32 healthy controls. Raw count data were obtained and processed using the DESeq2 R package. Following normalization using the variance stabilizing transformation (VST), we conducted differential expression analysis comparing severe AP patients against healthy controls. Genes were considered significantly differentially expressed at FDR adjusted *p*‐value < 0.05. We specifically examined the expression patterns of our high‐confidence pleiotropic genes within this cohort to determine their association with disease severity.

### 2.8 Genetically Informed Spatial Mapping of Pleiotropic Genes

To elucidate the spatial architecture of genetic risk for acute pancreatitis, we implemented the gsMap (genetically informed spatial mapping of cells for complex traits) algorithm, a powerful framework recently introduced in *Nature* for integrating GWAS data with spatial transcriptomics (ST) [8]. This approach integrates GWAS summary statistics with spatially resolved single‐cell transcriptomic data to map disease‐associated cellular patterns at single‐cell resolution. We analyzed ST data from an alcohol‐ and cerulein‐induced acute pancreatitis mouse model, comprising pancreatic FFPE sections from young (*n* = 2) and aging (*n* = 2) C57BL/6 male mice [9]. Based on canonical marker gene expression and morphological features, we annotated distinct cellular compartments, including acinar, ductal, endocrine, immune, and mesothelial cells, as well as inflammatory‐activated cells and macrophages. The gsMap algorithm was applied by first calculating gene‐specific scores (GSS) for each high‐prioritized pleiotropic gene. Spatial coordinates were preserved to maintain tissue architecture information. A high GSS indicates that a gene's expression is highly concentrated in a particular niche. This score was then assigned to all SNPs within a 100 kb window, functionally annotating each SNP with the spatial expression profile of its potential target gene in the context of pancreatitis. Human orthologs were mapped using biomaRt (version 2.50.3) with stringent quality filters.

### 2.9 Target Prioritization and In Silico Repurposing

A computational pipeline was established to identify and prioritize drugs for repurposing against the comorbidity of AP and infections. Our primary strategy involved a systematic, pathway‐based alignment of drug action with disease pathology. The set of high‐confidence pleiotropic genes was cross‐referenced with the DrugCentral, DGIdb, and PharmGKB databases to identify interacting drugs. Key pathological pathways disrupted by the pleiotropic genes were then defined using enrichment analysis via ClusterProfiler. In parallel, the pharmacological pathways modulated by each candidate drug were identified based on their known targets. A quantitative pairing score was subsequently calculated to measure the concordance between a drug's pharmacological action and the disease's pathophysiology, prioritizing drugs with a high degree of overlap as promising repurposing candidates. For the specific methodology of the pairing score, please refer to the research by Gao et al. [10] As a complementary, direct‐lookup approach, we also queried the DrugBank database to rapidly identify any established drugs known to target the pleiotropic genes.

## 3 Results

### 3.1 GWAS Data and Quality Control

We obtained GWAS summary statistics for AP, which comprised over 19 million SNPs, and for 17 infectious diseases, each with over 21 million SNPs. Detailed information on these cohorts, including sample sizes and data sources, is provided in Table S1. The quality and statistical power of each GWAS were rigorously assessed by estimating SNP‐based heritability using LDSC. Our quality control protocol required a trait's heritability estimate to be statistically significant. We found that eight of the infectious disease traits (Candidiasis, Central nervous system inflammatory conditions, *C. difficile* enterocolitis, CMV infection, Herpes zoster, Tuberculosis, Mycoses, Infectious Mononucleosis, and Streptococcal Septicemia) exhibited low and non‐significant SNP heritability. Specifically, these traits had heritability *Z*‐scores < 3 with corresponding *p*‐values approaching or exceeding 0.05. This indicates that the null hypothesis of zero heritability could not be rejected, suggesting a weak polygenic signal in these datasets. In contrast, AP and the remaining eight infectious disease traits demonstrated robust and significant SNP‐based heritability, with all *Z*‐scores substantially greater than 3. Notably, ATIN, Cystitis, and SSI showed the strongest heritability signals. Furthermore, the LDSC intercepts for all retained traits were approximately 1.0 and did not deviate significantly from this value, suggesting that confounding biases such as population stratification were minimal and well‐controlled. A summary of all LDSC results is presented in Table S2.

### 3.2 Genetic Correlation and Causal Inference

**FIGURE 2:**
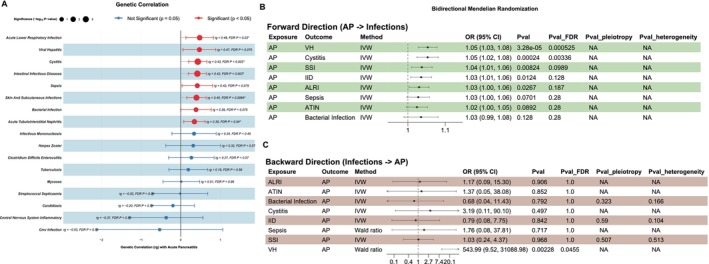
Genetic correlation and bidirectional causal inference between acute pancreatitis (AP) and Infectious Diseases. (A) Forest plot displaying the genetic correlations (rg) between AP and nine infectious diseases. (B) Results of forward two‐sample Mendelian randomization (MR) assessing the causal effect of AP on each infectious disease. (C) Results of reverse‐direction MR assessing the causal effect of each infectious disease on AP.

We performed LDSC regression for each AP and infectious trait pair, identifying five pairs with statistically significant positive genetic correlations after correction for multiple testing: ALRI (rg = 0.48, *p*_FDR_ = 0.03), Cystitis (rg = 0.43, *p*_FDR_ = 0.003), IID (rg = 0.42, *p*_FDR_ = 0.003), SSI (rg = 0.40, *p*_FDR_ = 0.0084), and ATIN (rg = 0.35, *p*_FDR_ = 0.04). Additionally, several other traits showed nominally significant positive correlations, including VH (rg = 0.47), sepsis (rg = 0.42), and bacterial infection (rg = 0.39). Although these associations did not remain significant after FDR correction (*p*_FDR_ > 0.05), they were retained for subsequent genetic analysis due to their strong correlation coefficients and clinical relevance (Figure 2A). To investigate whether these observed genetic correlations represent causal relationships, we conducted bi‐directional two‐sample Mendelian Randomization. In the forward‐direction analysis, a genetically predicted liability to AP was nominally associated with an increased risk of five infectious diseases: VH (OR: 1.05, 95% CI: 1.03–1.08; *p* = 3.28 × 10^−5^), Cystitis (OR: 1.05, 95% CI: 1.02–1.08; *p* = 0.24 × 10^−3^), SSI (OR: 1.04, 95% CI: 1.01–1.06; *p* = 0.008), IID (OR: 1.03, 95% CI: 1.01–1.06; *p* = 0.0124), and ALRI (OR: 1.03, 95% CI: 1.00–1.06; *p* = 0.0267). After applying FDR correction, the causal effects on VH (*p*_FDR_ = 0.525 × 10^−3^) and Cystitis (*p*_FDR_ = 0.003) remained statistically significant (Figure 2B). In the reverse‐direction analysis, a genetic predisposition to VH showed a significant causal association with an increased risk of AP (OR: 543.99, 95% CI: 9.52–31088.98; *p* = 0.002), which also remained significant after FDR correction (*p*_FDR_ = 0.0455). No other infectious traits showed a nominally significant causal effect on AP risk (Figure 2C). Importantly, sensitivity analyses for these MR tests revealed no significant directional pleiotropy or heterogeneity, supporting the robustness of the causal estimates.

### 3.3 Cross‐Trait Analysis and Identification of Pleiotropic Variants

We performed a cross‐trait meta‐analysis using MTAG for AP and eight infectious disease traits: ALRI, Cystitis, IID, SSI, ATIN, VH, sepsis, and bacterial infection. To specifically test for pleiotropy while accounting for potential heterogeneity, this was complemented by CPASSOC analysis. Applying a stringent significance threshold (*p*_MTAG_ < 5 × 10^−8^ and *p*_CPASSOC_ < 5 × 10^−8^), we identified a total of 108 unique pleiotropic variants across the eight trait pairs. The number of variants identified per pair ranged from 18 to 65, with the AP‐ATIN analysis yielding the most loci (Figure S1). Functional annotation using ANNOVAR revealed that three (2.8%) of the pleiotropic variants were located in exonic regions, including rs1800947 (*CRP*), rs17107315 (*SPINK1*), and rs7853989 (*ABO*). The majority of the remaining variants (91.7%) were located in intronic or intergenic regions (Figure S2; Table S3). To prioritize likely causal variants from these signals, we then performed Bayesian fine‐mapping on the surrounding genomic regions (±500 kb). This analysis refined 231 variants within these regions into a 99% credible set containing 141 putative causal SNPs. Among these, we identified nine high‐confidence pleiotropic variants: rs1800947 (*CRP*), rs74596724 (*CRP*), rs559363229 (*JAKMIP2; SPINK1*), rs2073823 (*ABO*), rs7853989 (*ABO*), rs8176722 (*ABO*), rs8176725 (*ABO*), rs8176730 (*ABO*), and a second independent signal for rs559363229 (*JAKMIP2; SPINK1*). Notably, with the exception of the two *CRP* variants, which are located on chromosome 1 and exhibit positive effect sizes, the remaining seven variants are located on either chromosome 5 or 9 and all exhibit negative effect sizes (Figure S3).

### 3.4 Pleiotropic Genes Identification

**FIGURE 3:**
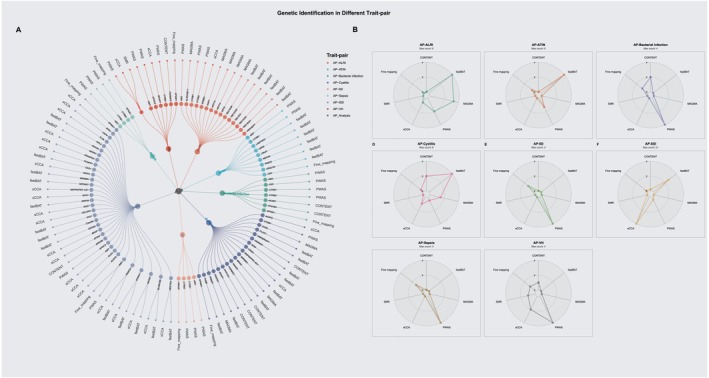
Multi‐omic identification of candidate pleiotropic genes. (A) Circular plots illustrating the number of significant pleiotropic genes identified by each of the seven analytical methods for each AP‐infection trait pair. (B) Radar charts showing the number of significant genes identified by each omic‐level analysis after FDR correction.

**FIGURE 4:**
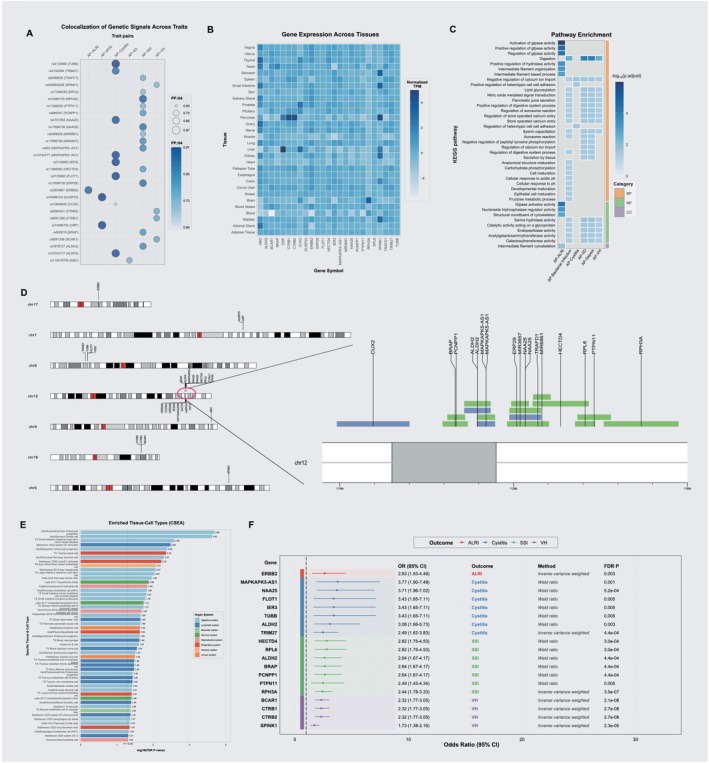
Functional characterization and causal inference for high‐confidence pleiotropic genes. (A) Summary of Bayesian colocalization results (PPH_4_) for candidate genes. (B) Heatmap of normalized expression for high‐confidence genes across various human tissues. (C) Results of pathway enrichment analysis. (D) Ideogram showing the chromosomal locations of the high‐confidence genes. (E) Results of cell‐type specific enrichment analysis (CSEA). (F) Forest plot of locus‐specific MR results testing the causal effects of gene expression on disease risk.

To identify candidate effector genes for the pleiotropic loci, we implemented a multi‐omic analytical strategy integrating transcriptomic, proteomic, and single‐cell level data. At the transcriptomic level, a cross‐tissue analysis using sCCA identified 24 pleiotropic genes after FDR correction, with the majority discovered in the AP‐SSI trait pair (Table S4; Table S5). As complementary approaches, two gene‐based association tests that aggregate SNP‐level signals were performed. These tests implicated further candidates, with GCTA‐fastBAT identifying 30 unique genes and MAGMA identifying 8 unique genes significantly associated with AP‐infection trait pairs (Tables S6 and S7). Focusing on tissue‐specific effects, we identified a significant regulatory link in the pancreas, where the SNP rs8057145 is associated with the expression of *CTRB2*, influencing the risk of both AP and associated infections. To resolve effects at the cellular level, we applied CONTENT, a method that decomposes gene expression into context‐shared and context‐specific components to enhance statistical power. This analysis revealed significant expression associations for eight genes within four specific immune cell populations including plasmacytoid dendritic cells (pDCs), CD8+ T cells, CD4+ T cells, and non‐classical monocytes (Table S8), highlighting a potential cellular basis for the observed pleiotropy. At the proteomic level, a PWAS found that the genetically predicted levels of five unique plasma proteins—*ABO*, *CTRB1*, *CTRB2*, *CRP*, and *APOC3*—were significantly associated with the risk of eight infectious diseases (Table S9). This finding provides evidence for shared pathogenic pathways manifesting at the protein level. A comprehensive summary of all identified pleiotropic genes, with counts per methodology, is detailed in Figure 3A and Figure 3B. To validate whether the 61 unique genes previously identified represent true shared genetic associations, we performed Bayesian colocalization analysis for each corresponding trait pair. Following this stringent analysis, 29 genes showed strong evidence of harboring a shared causal variant between AP and an infectious disease, defined by a posterior probability (PPH_4_) greater than 0.6 (Figure 4A; Table 1). This high‐confidence set of 29 genes formed the basis for all subsequent functional interrogation.

**TABLE 1:** Bayesian colocalization of pleiotropic loci for acute pancreatitis and infectious diseases.

Cross trait	Genomic region	Gene	SNP	PPH4	PPH0	PPH1	PPH2	PPH3	Analysis
AP‐ALRI	17‐39637914‐39780426	ERBB2	rs2934967	0.897	0	0.001	0.016	0.086	sCCA
AP‐ATIN	1‐159693672‐159793672	CRP	rs74596724	0.968	0	0	0.03	0.002	Fine‐mapping
AP‐ATIN	1‐159663648‐159763648	DUSP23	rs74596724	0.967	0	0	0.031	0.002	Fine‐mapping
AP‐Cystitis	6‐30667435‐30775538	TUBB	rs3130660	0.972	0	0	0.005	0.022	fastBAT
AP‐Cystitis	6‐30677709‐30792732	FLOT1	rs3130660	0.964	0	0	0.005	0.03	fastBAT
AP‐Cystitis	6‐30693199‐30794548	IER3	rs3130660	0.963	0	0.001	0.006	0.031	fastBAT
AP‐Cystitis	12‐111789758‐111892902	MAPKAPK5‐AS1	rs10744777	0.956	0.021	0.007	0.013	0.003	sCCA
AP‐Cystitis	12‐111716887‐111867532	ALDH2	rs10744777	0.951	0.011	0.01	0.015	0.013	sCCA
AP‐Cystitis	12‐111976689‐112158796	NAA25	rs4767293	0.937	0.033	0.005	0.022	0.003	fastBAT
AP‐Cystitis	6‐28853002‐28973988	TRIM27	rs3132384	0.88	0.004	0.004	0.057	0.055	MAGMA
AP‐Cystitis	12‐110984165‐111400554	CUX2	rs10849943	0.605	0.211	0.032	0.133	0.02	MAGMA
AP‐IID	9‐133183278‐133326024	ABO	rs115478735	0.732	0.002	0.001	0.208	0.057	PWAS
AP‐SSI	12‐112520380‐112948881	RPH3A	rs12580178	0.915	0.042	0.017	0.019	0.007	fastBAT
AP‐SSI	12‐111963348‐112073449	ERP29	rs17696736	0.875	0.027	0.014	0.055	0.028	fastBAT
AP‐SSI	12‐111976689‐112158796	NAA25	rs17696736	0.866	0.036	0.017	0.057	0.025	fastBAT, sCCA
AP‐SSI	12‐111789758‐111892902	MAPKAPK5‐AS1	rs440	0.825	0.011	0.015	0.064	0.085	sCCA
AP‐SSI	12‐111716887‐111867532	ALDH2	rs7978737	0.819	0.01	0.018	0.057	0.096	sCCA
AP‐SSI	12‐111987599‐112087715	MIR3657	rs17696736	0.808	0.094	0.021	0.063	0.014	fastBAT
AP‐SSI	12‐111592146‐111735956	BRAP	rs630616	0.795	0.055	0.046	0.057	0.047	sCCA
AP‐SSI	12‐111619852‐111720362	PCNPP1	rs695047	0.782	0.081	0.048	0.057	0.033	sCCA
AP‐SSI	12‐112110188‐112432439	HECTD4	rs11066283	0.776	0.093	0.028	0.079	0.023	fastBAT, sCCA
AP‐SSI	12‐112075538‐112203604	TRAFD1	rs6489828	0.731	0.177	0.023	0.061	0.007	fastBAT, sCCA
AP‐SSI	12‐112368351‐112559918	PTPN11	rs11066283	0.714	0.156	0.03	0.085	0.016	fastBAT, sCCA
AP‐SSI	12‐112113258‐112213321	MIR6861	rs6489828	0.706	0.215	0.021	0.054	0.005	fastBAT
AP‐SSI	12‐112355189‐112468838	RPL6	rs11066283	0.696	0.191	0.032	0.07	0.011	fastBAT
AP‐VH	16‐75168988‐75276338	CTRB1	rs8061356	0.757	0.013	0.208	0.001	0.02	PWAS
AP‐VH	16‐75178181‐75318053	BCAR1	rs8061356	0.756	0.013	0.208	0.001	0.021	sCCA
AP‐VH	16‐75154103‐75257161	CTRB2	rs6564241	0.746	0.016	0.219	0.001	0.017	PWAS, sCCA, SMR
AP‐VH	5‐147755265‐147855265	SPINK1	rs559363229	0.712	0	0.278	0	0.011	Fine‐mapping

Abbreviations: ALRI, acute lower respiratory infection; AP, acute pancreatitis; ATIN, acute tubulointerstitial nephritis; IID, intestinal infectious diseases; SSI, skin and subcutaneous infections; VH, viral hepatitis.

An investigation into the tissue‐specific expression patterns of these 29 high‐confidence genes revealed significant enrichment in the pancreas (e.g., *ABO, CRP, CTRB1, CTRB2, SPINK1*) and liver (e.g., *CRP, CUX2, SPINK1*), suggesting these are primary sites of their biological function (Figure 4B). This was further supported by differential expression analysis, which showed a significant collective upregulation of this gene set in pancreatic tissue (Figure S4). Subsequent pathway enrichment analysis highlighted significant overrepresentation in biological processes such as “Activation of GTPase activity,” “Digestion,” and molecular functions including “Serine hydrolase activity” (Figure 4C). Chromosomal mapping showed a notable clustering of these genes on chromosome 12, with smaller groups located on chromosomes 6 and 16 (Figure 4D). Cell‐type specific enrichment analysis (CSEA) revealed that the pleiotropic genes were significantly enriched across a diverse range of cell types. The analysis strongly implicated cells of the digestive system, with the most significant enrichment observed in Adult Ascending Colon Enterocyte progenitors, Adult Stomach Goblet cells, and crucial pancreatic cell types such as Pancreas exocrine cells and Fetal Pancreas Acinar cells. Furthermore, multiple immune cell populations were also significantly enriched, including classical monocytes, macrophages, and dendritic cells. These findings indicate that the shared genetic pathways linking AP and infection are active in both digestive/pancreatic tissues and key components of the immune system (Figure 4E). Finally, to identify which of these genes represent potentially druggable targets, we performed a locus‐specific MR analysis. After correction for multiple testing, this analysis revealed that the genetically predicted expression of all 18 tested genes had a significant causal association with at least one of the analyzed traits. Notable examples include *NAA25* (OR = 3.71, 95% CI: 1.95–7.02, *p*_FDR_ = 5.2 × 10^−4^) and *BCAR1* (OR = 2.32, 95% CI: 1.77–3.05, *p*_FDR_ = 2.1 × 10^−8^) (Figure 4F). These convergent findings from multiple lines of bioinformatic evidence underscore the robustness of these pleiotropic genes as key mediators of the comorbidity between AP and infectious diseases.

### 3.5 Peripheral Blood Transcriptomic Validation in Human Acute Pancreatitis Patients With Infection

**FIGURE 5:**
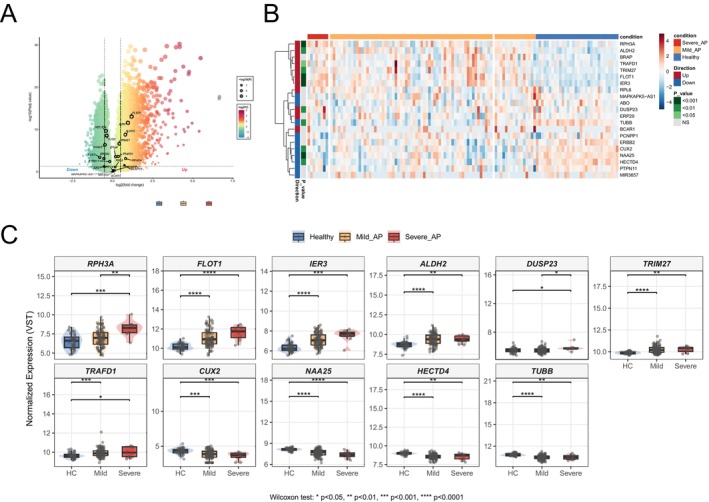
Peripheral blood transcriptomic validation of pleiotropic genes in human acute pancreatitis. (A) Volcano plot showing differential gene expression in peripheral blood from acute pancreatitis patients compared to healthy controls. The *x*‐axis represents log2(fold change) and the *y*‐axis represents −log10(adjusted *p*‐value). Genes with log2FC > 0.5 and FDR‐adjusted *p* < 0.05 are highlighted in red (upregulated) or blue (downregulated). (B) Heatmap displaying hierarchical clustering of pleiotropic gene expression profiles across individual samples. Rows represent the 29 high‐confidence pleiotropic genes, columns represent individual samples grouped by disease severity. (C) Box plots showing normalized expression levels (VST) of 11 representative pleiotropic genes across disease severity groups. Statistical significance determined by Wilcoxon rank‐sum test with Bonferroni correction: *p < 0.05, **p < 0.01, ***p < 0.001, ****p < 0.0001.

Bulk peripheral‐blood transcriptomes showed that, relative to healthy controls, patients with AP exhibited marked differential expression of genes including FLOT1, IER3, HECTD4, TUBB0, TRIM27, and ALDH2 (Figure 5A). Specifically, a distinct set of genes, including *FLOT1*, *IER3*, *ALDH2*, *RPH3A*, and *DUSP23*, was significantly upregulated in the peripheral blood of AP patients, satisfying the criteria of a log2(fold change (FC)) > 0.5 and an FDR‐adjusted *p*‐value < 0.05. Other genes, including *TRIM27*, *BRAP*, and *TRAFD1*, demonstrated a trend towards upregulation but did not meet the statistical significance (Figure 5A,B). Critically, the expression levels of several of these pleiotropic genes correlated with disease severity, a clinically relevant finding as increased severity is strongly associated with the development of infection. We observed a stepwise, severity‐dependent increase in the expression of *FLOT1*, *IER3*, *RPH3A*, and *DUSP23* whereas ALDH2 and TRIM27 showed no significant differences between mild and severe disease (Figure 5C).

### 3.6 Genetically Informed Spatial Mapping

**FIGURE 6:**
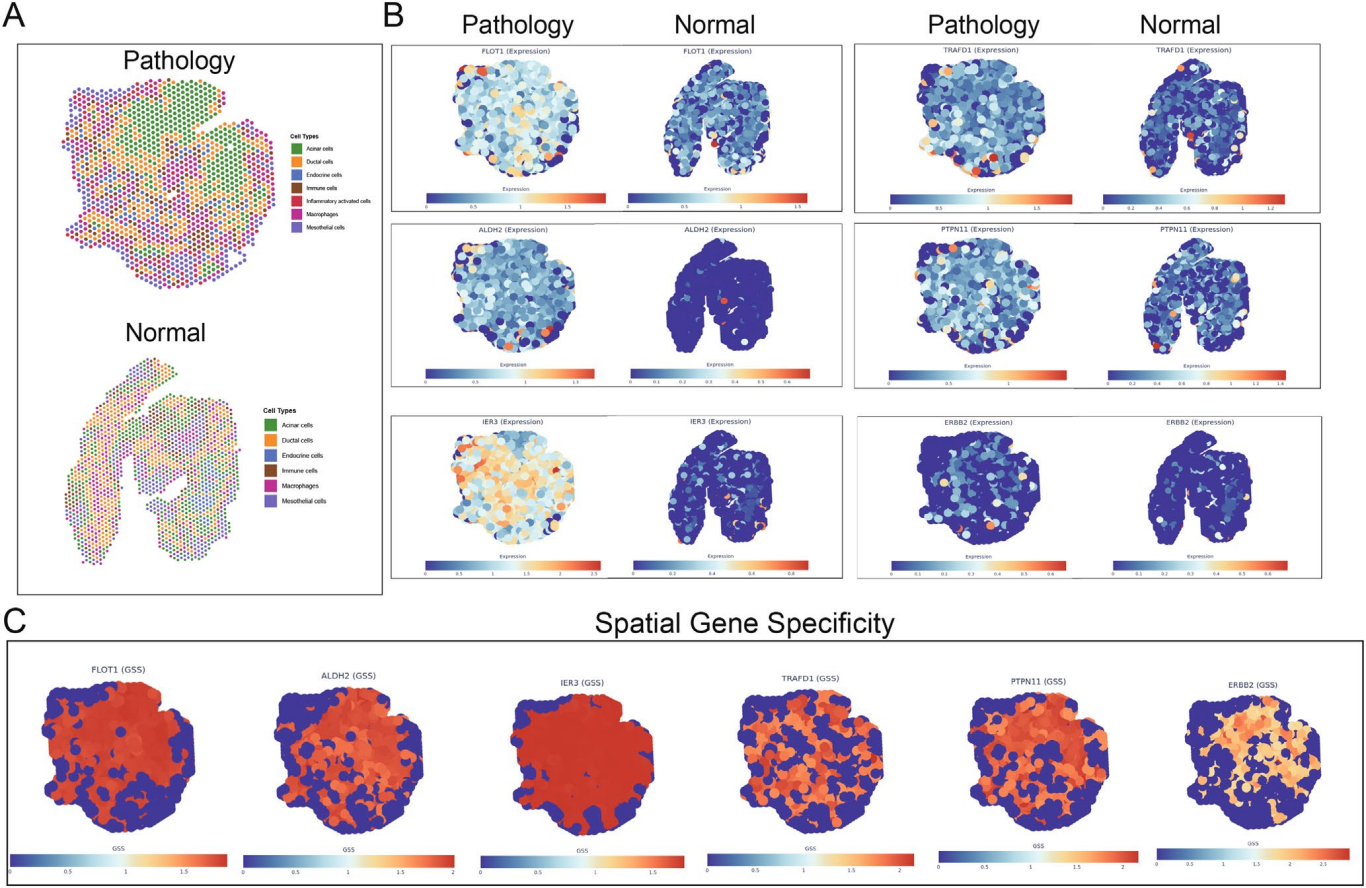
Spatially resolved expression mapping of pleiotropic genes in murine acute pancreatitis. (A) Spatial transcriptomic analysis of tissue architecture in a murine model of pathological acute pancreatitis compared to normal pancreatic tissue. Each dot represents a spatial spot colored by cell type annotation based on canonical marker expression. (B) Spatial expression patterns of six key pleiotropic genes comparing pathological (left) and normal (right) pancreatic tissue. Expression levels are visualized using a gradient color scale from low (blue) to high (red). (C) Gene‐specific score (GSS) analysis quantifying spatial expression specificity for each pleiotropic gene. Higher GSS values (red) indicate concentrated expression in specific tissue niches, while lower values (blue) represent diffuse expression. GSS calculations were performed using the gsMap algorithm with 100 kb flanking windows.

Application of the gsMap algorithm to spatial transcriptomic data from murine acute pancreatitis revealed heterogeneity in the spatial distribution of pleiotropic gene expression across pancreatic tissue architecture. The GSS, which quantifies the relative expression specificity of each gene within distinct spatial compartments, demonstrated that pleiotropic genes associated with AP‐infection comorbidity are not randomly distributed but exhibit compartmental localization reflecting their functional roles in disease pathogenesis. We first utilized pathological and normal pancreata ST data to perform regional mapping based on canonical marker genes. Using established pancreatic cell type markers from the Human Protein Atlas and PanglaoDB databases, combined with dimensionality reduction and unsupervised clustering, we annotated distinct cellular compartments within the ST data. Figure 6A revealed reorganization of cellular architecture during pancreatitis. The pathological sections showed disruption of normal acinar organization, with the emergence of distinct cellular compartments including stressed acinar cells, inflammatory‐activated cells, ductal proliferation zones, and areas of mesenchymal expansion. Normal tissue maintained organized acinar structure with minimal inflammatory infiltration and intact ductal architecture. We assessed the association of pancreatic regions with AP‐infection risk by aggregating *p* values of individual cells in each region using the Cauchy combination test. The acinar cells (*p* = 2.3 × 10^−5^), ductal cells (*p* = 4.1 × 10^−4^), and inflammatory‐activated cells (*p* = 8.7 × 10^−5^) showed the highest relevance to infection susceptibility. Figure 6B demonstrated differential expression patterns of key pleiotropic genes between normal and pathological tissue. *FLOT1, ALDH2* and *TRAFD1* showed markedly increased expression in diffuse pathological tissue compared to normal tissue. *IER3* exhibited the most dramatic change, with high expression observed throughout pathological tissue while remaining nearly absent in normal samples. *PTPN11* demonstrated elevated expression primarily in stressed acinar cells and inflammatory zones of pathological tissue versus normal tissue. *ERBB2* showed preferential upregulation in ductal cell regions of pathological samples compared to normal status. Figure 6C illustrated the spatial gene specificity patterns using GSS calculations across tissue compartments. *IER3* demonstrated the highest spatial restriction among analyzed genes, with expression concentrated in specific inflammatory microdomains. *FLOT1* and *TRAFD1* showed high spatial specificity, while *ALDH2* and *PTPN11* displayed intermediate specificity patterns. *ERBB2* demonstrated more diffuse expression with lower spatial restriction.

### 3.7 Drug Class Signals From the In Silico Screen

To identify potential therapeutic candidates, we implemented a systematic drug repurposing pipeline centered on the 29 high‐confidence pleiotropic genes. This approach utilized a “pairing score” to quantify the alignment between the known pharmacological pathways of existing drugs and the pathological pathways disrupted by the pleiotropic genes. This pathway‐based method offers greater mechanistic insight than simple drug‐gene interaction lookups. The results of this pairing analysis revealed several drug classes with high scores across multiple AP‐infection trait pairs, notably HER/EGFR Inhibitors, ROCK Inhibitors, and S1P Receptor Modulators (Figure S5). Among the top individual candidates were the ROCK inhibitor Sovesudil and the S1P Receptor Modulator Ponesimod. As a complementary approach, we performed a direct search of the DRUGBANK database for compounds targeting key pleiotropic genes. For *ALDH2*, this search identified Guanidine, Daidzin, and Disulfiram as compounds with established pharmacological actions. Two other related molecules, NADH and Crotonaldehyde, were also linked to *ALDH2*, but their specific pharmacological actions have not been determined.

## 4 Discussion

AP extends beyond a localized disorder of pancreatic acinar cells, with extrapancreatic and extrahepatic factors playing crucial roles in disease pathogenesis. Previous large‐scale GWAS meta‐analyses of AP have demonstrated that proteome‐wide Mendelian randomization reveals susceptibility genes including *ABCG5/G8* and *SPINK1*, highlighting the potential for genetic exploration of therapeutic targets and preventive strategies for AP. Up to exceeding 20% of acute pancreatitis patients develop extrapancreatic infections, including bloodstream infections, pneumonia, and urinary tract infections. Infection represents a critical culprit in the development of persistent organ failure. However, clinically, infection accompanies the entire treatment course of AP, making the diagnosis of concurrent infection particularly challenging. This diagnostic difficulty arises because patients with severe acute pancreatitis (SAP) present with severe inflammatory responses at disease onset, including high fever, markedly elevated white blood cell counts, dramatically increased CRP and procalcitonin levels, multi‐organ dysfunction, and even shock. The clinical manifestations of SAP are difficult to distinguish from sepsis and septic shock, leading to inappropriate antibiotic usage in clinical practice. Furthermore, due to complex bacterial resistance patterns, many AP patients cannot promptly receive effective antibiotics, resulting in delayed treatment and significant challenges in clinical AP patient management [11].

Our post‐GWAS investigation identified moderate genetic correlations between AP and a range of infectious diseases, with genetic correlation coefficients ranging from 0.35 to 0.48. The identification of several high‐confidence pleiotropic genes with well‐documented roles in pancreatic pathophysiology provides a robust biological foundation for our findings. The discovery of *SPINK1* is particularly noteworthy, given its established role in preventing premature, trypsin‐catalyzed activation of zymogens within the pancreas [12, 13, 14]. As mutations in *SPINK1*, especially the N34S variant, are critical genetic risk factors for chronic pancreatitis [12, 14], its dual function in pancreatic cytoprotection and immune regulation suggests that its genetic variants may simultaneously compromise local pancreatic defense and systemic antimicrobial responses [13]. The identification of CRP, a classic acute‐phase reactant, as a pleiotropic gene aligns perfectly with its clinical utility as a prognostic biomarker for AP severity [15]. *IER3* was similarly highlighted, corroborating prior reports [16, 17]. Our transcriptomic validation in human patients reinforced this, demonstrating a severity‐dependent upregulation of *IER3* in peripheral blood. Furthermore, our spatial analysis demonstrated that its expression was markedly elevated in inflammatory microdomains, identifying it as a pivotal regulator of the acute inflammatory response. Our locus‐specific MR analysis demonstrated that elevated *IER3* expression significantly increases the risk of cystitis in patients with AP (OR 3.43, 95% CI 1.65–7.11), reinforcing its role in acute inflammation. Furthermore, the identification of the Chymotrypsinogen B1/B2 (*CTRB1*/*CTRB2*) locus underscores the importance of digestive enzymes in both local pancreatic homeostasis and systemic immunity, consistent with preclinical evidence showing that chymotrypsin mitigates pancreatitis severity by degrading trypsin [18, 19].

Beyond previously established susceptibility loci, our study identified several novel AP risk loci strongly associated with infection risk. The pleiotropy of the *ABO* gene offers evidence for the role of innate immunity at the AP‐infection interface, consistent with previously documented associations between *ABO* polymorphisms and susceptibility to infectious diseases, pancreatic cancer, and AP. The discovery of *ERBB2* (*HER2*), a receptor tyrosine kinase well‐studied in pancreatic cancer [20], provides a new perspective on AP‐infection comorbidity, suggesting a complex regulatory function in inflammation [21]. Our spatial analysis reveals a pathological redistribution of *ERBB2* during pancreatitis progression, with significant upregulation and enrichment in the ductal and peri‐ductal stromal regions of pathological tissue. Our investigation also extended to genes governing fundamental cellular processes. The identification of *FLOT1* and *TUBB* indicates that cytoskeletal organization and membrane trafficking may contribute to the interplay between AP and infection. Bulk transcriptomic analysis of peripheral blood from patients provided initial clinical support for this association, showing that both genes were significantly upregulated during AP. Expression of *FLOT1*, in particular, increased progressively with disease severity, suggesting a potential involvement in the systemic inflammatory response. Spatial transcriptomic analysis further contextualized these findings by localizing *FLOT1* expression within regions of dense immune cell infiltration in injured pancreatic tissue, implying a site‐specific role in modulating local inflammation. This spatially anchored observation complements our locus‐specific Mendelian randomization results, which suggested that higher expression levels of *FLOT1* and *TUBB* are causally associated with an elevated risk of urinary tract infection.

Further pleiotropic loci implicated cellular regulation and metabolism. The rs632650 T>G variant near *PCNPP1* and *RPH3A* was associated with increased risk of both AP and infection, and colocalization analysis showed the rs695047 variant in the *PCNPP1* locus was shared between AP and SSI. Furthermore, this was supported by transcriptomic data showing significant, severity‐dependent upregulation of RPH3A in patients. *RPH3A* has a known role in neurodevelopmental disorders [22]. Variants in *ALDH2*, a key enzyme in alcohol metabolism, were also shared between AP and SSI. Increased *ALDH2* expression was causally linked to a higher probability of SSI, suggesting a pleiotropic mechanism may be relevant to alcoholic pancreatitis via oxidative stress pathways [23]. Our spatial analysis supports this genetic finding, showing pronounced upregulation of *ALDH2* in damaged acinar cells that are likely experiencing high levels of oxidative stress. This feature was further delineated in the transcriptomic analysis of peripheral blood, and subsequently corroborated in a meta‐analysis using an allelic contrast model [24]. We also identified *NAA25* and *RPL6*, enzymes crucial for co‐translational N‐terminal acetylation. Their dysregulation, often observed in cancer, can trigger endoplasmic reticulum stress—a pivotal mechanism in pancreatitis pathogenesis [25, 26]. *BCAR1* which modulates cell adhesion, migration, and proliferation, a signaling adaptor protein, showed a significant causal association with VH infection (OR = 2.32, 95% CI 1.77–3.05). At the 12q24 locus, the rs11066283 variant—an eQTL associated with elevated *PTPN11* expression in blood—was found in our analyses to be linked with an increased risk of AP and secondary infection. This association is biologically plausible, considering SHP2's well‐established role in modulating cytokine receptor signaling and JAK–STAT pathway activity. Spatial transcriptomic profiling of AP tissue further supported this observation, revealing a significant upregulation of both *PTPN11* and *TRAFD1* within acinar regions and immune cell–rich inflammatory infiltrates. The regulatory roles of the lncRNA *MAPKAPK5‐AS1* and the ubiquitin ligase genes *HECTD4* and *TRIM27* were also confirmed, with risk‐associated variants appearing to alter key immune protein regulation, thereby contributing to the pathogenesis of both AP and subsequent infections. Notably, HECTD4 expression was reduced in peripheral blood during AP, suggesting a possibly dysregulated immune response that may impair the ability to combat infections during AP progression.

Building on these mechanistic insights, our study provides a framework for generating data‐driven hypotheses for drug repurposing. Our computational screen highlighted several therapeutic classes, including HER2/EGFR inhibitors, Rho Kinase (ROCK) inhibitors, and S1P Receptor Modulators, as promising candidates due to their association with the key pleiotropic pathways identified. The prioritization of HER2/EGFR inhibitors is particularly compelling, as it converges with our spatial transcriptomic data demonstrating the pathological redistribution of ERBB2 to regions of ductal remodeling, providing a strong mechanistic rationale for testing these agents to preserve barrier integrity. Similarly, the relevance of ROCK inhibitors is underscored by the known role of the Rho/ROCK signaling pathway as a critical mediator of both cytoskeletal integrity and the inflammatory responses seen in sepsis and its sequelae [27]. Likewise, S1P Receptor Modulators such as Fingolimod are known for their efficacy in various inflammatory conditions [28]. Although animal models suggest these agents can mitigate complications of pancreatitis, they are not yet widely studied in this context. Nevertheless, the computational signals reported here should be regarded as hypothesis‐generating, not as evidence of clinical efficacy, and they serve to prioritize candidates for mechanistic and preclinical testing.

While this study provides the most comprehensive investigation to date of the shared genetic architecture between acute pancreatitis and infectious diseases, several limitations should be acknowledged. First, although our computational findings are now supported by experimental transcriptomic and spatial validation, the murine spatial transcriptomics model may not fully recapitulate all aspects of human disease heterogeneity. Therefore, the identified pleiotropic loci, causal inferences, and prioritized drug candidates represent statistical predictions that require experimental validation in relevant cellular and animal models to confirm their biological function. Second, a significant limitation of our study is that the GWAS summary statistics were predominantly sourced from cohorts of European ancestry. This reliance may limit the generalizability of our findings, as allele frequencies and linkage disequilibrium patterns can differ substantially across ancestries. A prime example is the *ALDH2* gene, where the rs671 (Glu504Lys) variant, common in East Asian populations and strongly associated with alcohol metabolism and pancreatitis risk, is virtually absent in European populations [29, 30]. Consequently, our study may have missed population‐specific risk loci like this one, while some identified associations may be ancestry‐specific. Therefore, we strongly advocate for and highlight the critical need for future large‐scale GWAS of acute pancreatitis and infectious diseases in diverse, non‐European cohorts (e.g., East Asian, African, and Hispanic populations) to uncover a more complete and globally applicable genetic architecture. Third, while MR is a powerful tool for inferring causality, it is predicated on assumptions that cannot be fully proven, such as the absence of horizontal pleiotropy. Although we performed multiple sensitivity analyses to mitigate this, the potential for unmeasured confounding remains. Finally, our drug repurposing pipeline is an in silico prioritization based on pathway alignment and curated target annotations rather than evidence of efficacy. Even with directionality checks, this approach does not account for pharmacokinetics and pharmacodynamics, toxicity, off‐target effects, or indication‐specific risks in infection‐prone settings, and it is limited by database incompleteness and the lack of cell‐state–specific pharmacology; accordingly, these outputs should guide mechanistic studies of target engagement and efficacy in pancreas‐relevant models rather than substitute for preclinical drug development.

In conclusion, this comprehensive post‐GWAS investigation successfully dissects the complex shared genetic landscape between acute pancreatitis and infectious diseases. By leveraging a multi‐layered analytical framework that moves beyond computational prediction to include functional validation, we identified 29 high‐confidence pleiotropic genes, including established loci like *SPINK1* and novel candidates such as *ERBB2*, *FLOT1*, and *PTPN11*. Crucially, we established a robust, multi‐modal evidence chain for their roles in pathogenesis. First, genetic and causal inference analyses confirmed them as shared risk factors. Second, bulk transcriptomic analysis of peripheral blood from human patients demonstrated their, severity‐dependent dysregulation, confirming their involvement in the systemic inflammatory response. Finally, by employing gsMap, a state‐of‐the‐art spatial GWAS mapping algorithm, we provided a spatially resolved mechanistic framework, pinpointing the activation of these genes to distinct pathological niches within the pancreas, such as inflammatory microdomains and zones of tissue remodeling. The convergence of genetic causality, systemic dysregulation in patients, and localized activation within diseased tissue underscores the pivotal role of these genes in the pathophysiology linking AP to infection. This is crucial for molecular‐level risk prevention in AP. Furthermore, it provides a solid foundation for prognostic stratification and early prevention, while simultaneously identifying and prioritizing validated therapeutic targets for future intervention.

## Author Contributions

Xing Chen, Shanshan Cai, and Xinglin Yi conceived and designed the study. Bo Zou and Daifeng Yang performed the data acquisition and statistical analyses. Bo Zou, Hao Jiang, and Jingsheng Ruan contributed to the interpretation of the data. Bo Zou and Shanshan Cai drafted the manuscript.

## Funding

The authors received no specific funding for this work.

## Consent

The authors have nothing to report.

## Conflicts of Interest

The authors declare no conflicts of interest.

## Supporting information


**Data S1:** jhbp70041‐sup‐0001‐Supinfo.docx.

## Data Availability

The authors have nothing to report.
