# Supplementary material for: Integrative Genomics Reveals Causal Pleiotropy and Therapeutic Opportunities at the Interface of Acute Pancreatitis and Infection
Source: J Hepatobiliary Pancreat Sci. 2025 Nov 27;33(3):194–207. doi: 10.1002/jhbp.70041 (PMC12993704; doi:10.1002/jhbp.70041)
Supplement: Supplementary file 1 — Data S1: jhbp70041‐sup‐0001‐Supinfo.docx. [file JHBP-33-194-s001.docx]

**Table S1.** Data source and description

| **Phenotype** | **Case** | **Control** | **Link** |
| --- | --- | --- | --- |
| **Acute pancreatitis** | 7613 | 439644 | http://ftp.ebi.ac.uk/pub/databases/gwas/summary_statistics/GCST90476001-GCST90477000/GCST90476106/GCST90476106.tsv.gz |
| **Sepsis** | 17133 | 439048 | <https://storage.googleapis.com/finngen-public-data-r12/summary_stats/release/finngen_R12_AB1_OTHER_SEPSIS.gz> |
| **Streptococcal Septicaemia** | 3239 | 439048 | <https://storage.googleapis.com/finngen-public-data-r12/summary_stats/release/finngen_R12_AB1_STREPTO_SEPSIS.gz> |
| **Clostridium Difficile Enterocolitis** | 4250 | 492689 | <https://storage.googleapis.com/finngen-public-data-r12/summary_stats/release/finngen_R12_C_DIFFICILE_ENTEROCOLITIS.gz> |
| **Candidiasis** | 5341 | 488977 | <https://storage.googleapis.com/finngen-public-data-r12/summary_stats/release/finngen_R12_AB1_CANDIDIASIS.gz> |
| **Intestinal Infectious Diseases** | 56056 | 444292 | <https://storage.googleapis.com/finngen-public-data-r12/summary_stats/release/finngen_R12_AB1_INTESTINAL_INFECTIONS.gz> |
| **Bacterial Infection** | 25506 | 439048 | <https://storage.googleapis.com/finngen-public-data-r12/summary_stats/release/finngen_R12_AB1_BACTINF_NOS.gz> |
| **Mycoses** | 11371 | 488977 | <https://storage.googleapis.com/finngen-public-data-r12/summary_stats/release/finngen_R12_AB1_MYCOSES.gz> |
| **Viral Hepatitis** | 2776 | 497572 | <https://storage.googleapis.com/finngen-public-data-r12/summary_stats/release/finngen_R12_AB1_VIRAL_HEPATITIS.gz> |
| **Infectious Mononucleosis** | 3838 | 486087 | <https://storage.googleapis.com/finngen-public-data-r12/summary_stats/release/finngen_R12_AB1_EBV.gz> |
| **Acute lower respiratory infection** | 6465 | 471806 | https://storage.googleapis.com/finngen-public-data-r12/summary_stats/release/finngen_R12_J10_ACUTELOWERNAS.gz |
| **Central nervous system inflammatory** | 3261 | 497087 | https://storage.googleapis.com/finngen-public-data-r12/summary_stats/release/finngen_R12_G6_NEUINFL.gz |
| **Tuberculosis** | 3063 | 497285 | https://storage.googleapis.com/finngen-public-data-r12/summary_stats/release/finngen_R12_AB1_TUBERCULOSIS.gz |
| **Herpes zoster** | 7132 | 480316 | https://storage.googleapis.com/finngen-public-data-r12/summary_stats/release/finngen_R12_AB1_ZOSTER.gz |
| **CMV Infection** | 604 | 499622 | https://storage.googleapis.com/finngen-public-data-r12/summary_stats/release/finngen_R12_CMV_NOS.gz |
| **Skin and subcutaneous infections** | 29841 | 470507 | https://storage.googleapis.com/finngen-public-data-r12/summary_stats/release/finngen_R12_L12_INFECT_SKIN.gz |
| **Cystitis** | 61460 | 403328 | https://storage.googleapis.com/finngen-public-data-r12/summary_stats/release/finngen_R12_N14_CYSTITIS.gz |
| **Acute Tubulointerstitial Nephritis** | 33228 | 447142 | https://storage.googleapis.com/finngen-public-data-r12/summary_stats/release/finngen_R12_N14_PYELONEPHR.gz |

**Table S2.** GWAS Quality Assessment

| **Trait** | **Chi-square** | **Intercept** | **Intercept standard error** | **SNP heritability** | **SNP heritability standard error** | **Z-score** | **SNP heritability Pval** |
| --- | --- | --- | --- | --- | --- | --- | --- |
| **Acute lower respiratory infection** | 1.0485 | 1.0055 | 0.0074 | 0.0046 | 0.0013 | 3.5773 | 0.0003 |
| **Acute Tubulointerstitial Nephritis** | 1.1216 | 1.0318 | 0.0074 | 0.0098 | 0.0011 | 8.9183 | 0.0000 |
| **Bacterial Infection** | 1.0654 | 1.0278 | 0.0071 | 0.0041 | 0.0012 | 3.3514 | 0.0008 |
| **Candidiasis** | 1.0382 | 1.0233 | 0.0061 | 0.0017 | 0.0009 | 1.8064 | 0.0709 |
| **Central nervous system inflammatory** | 1.0097 | 1.0029 | 0.0072 | 0.0007 | 0.0010 | 0.7183 | 0.4726 |
| **Clostridium Difficile Enterocolitis** | 1.0358 | 1.0190 | 0.0079 | 0.0018 | 0.0013 | 1.4218 | 0.1551 |
| **CMV Infection** | 1.0035 | 1.0018 | 0.0064 | 0.0002 | 0.0009 | 0.1957 | 0.8448 |
| **Cystitis** | 1.2269 | 1.0750 | 0.0075 | 0.0160 | 0.0015 | 10.8628 | 0.0000 |
| **Herpes zoster** | 1.0255 | 1.0122 | 0.0064 | 0.0014 | 0.0010 | 1.3289 | 0.1839 |
| **Infectious Mononucleosis** | 1.0486 | 1.0274 | 0.0072 | 0.0023 | 0.0011 | 2.0496 | 0.0404 |
| **Intestinal Infectious Diseases** | 1.1610 | 1.0334 | 0.0073 | 0.0140 | 0.0013 | 11.1703 | 0.0000 |
| **Mycoses** | 1.0438 | 1.0247 | 0.0062 | 0.0021 | 0.0010 | 2.0670 | 0.0387 |
| **Sepsis** | 1.0492 | 1.0172 | 0.0061 | 0.0035 | 0.0010 | 3.6408 | 0.0003 |
| **Skin and subcutaneous infections** | 1.1438 | 1.0274 | 0.0070 | 0.0128 | 0.0013 | 9.7047 | 0.0000 |
| **Streptococcal Septicaemia** | 1.0204 | 1.0102 | 0.0065 | 0.0010 | 0.0010 | 1.0833 | 0.2787 |
| **Tuberculosis** | 1.0209 | 0.9999 | 0.0072 | 0.0022 | 0.0011 | 2.0304 | 0.0423 |
| **Viral Hepatitis** | 1.0646 | 1.0227 | 0.0070 | 0.0045 | 0.0011 | 4.0732 | 0.0000 |
| **Acute Pancreatitis** | 1.0798 | 1.0462 | 0.0074 | 0.0038 | 0.0014 | 3.8034 | 0.0051 |

**Table S3.** Pleiotropic Variants in Each Trait Pair

| **SNP** | **Chrome** | **Position** | **Effect allele** | **Other allele** | **Consequence** | **Nearest Genes** | **Trait Pairs** |
| --- | --- | --- | --- | --- | --- | --- | --- |
| **rs12939565** | 17 | 39882136 | A | T | intergenic | ZPBP2(dist=4240),GSDMB(dist=22459) | AP-ALRI |
| **rs12939566** | 17 | 39882137 | A | T | intergenic | ZPBP2(dist=4241),GSDMB(dist=22458) | AP-ALRI |
| **rs62067029** | 17 | 39882138 | A | T | intergenic | ZPBP2(dist=4242),GSDMB(dist=22457) | AP-ALRI |
| **rs7359623** | 17 | 39893336 | C | T | intergenic | ZPBP2(dist=15440),GSDMB(dist=11259) | AP-ALRI |
| **rs869402** | 17 | 39911790 | T | C | intronic | GSDMB | AP-ALRI |
| **rs1011082** | 17 | 39912261 | T | C | intronic | GSDMB | AP-ALRI |
| **rs921650** | 17 | 39912823 | G | A | intronic | GSDMB | AP-ALRI |
| **rs921649** | 17 | 39913021 | C | T | intronic | GSDMB | AP-ALRI |
| **rs6503524** | 17 | 39913556 | C | T | intronic | GSDMB | AP-ALRI |
| **rs7216389** | 17 | 39913696 | C | T | intronic | GSDMB | AP-ALRI |
| **rs7216558** | 17 | 39913818 | C | T | intronic | GSDMB | AP-ALRI |
| **rs9303279** | 17 | 39917715 | G | C | intronic | GSDMB | AP-ALRI |
| **rs9303281** | 17 | 39917793 | G | A | intronic | GSDMB | AP-ALRI |
| **rs7219923** | 17 | 39918265 | C | T | intronic | GSDMB | AP-ALRI |
| **rs7224129** | 17 | 39919173 | G | A | upstream | GSDMB(dist=523) | AP-ALRI |
| **rs4065275** | 17 | 39924612 | A | G | intronic | ORMDL3 | AP-ALRI |
| **rs8076131** | 17 | 39924659 | G | A | intronic | ORMDL3 | AP-ALRI |
| **rs4795405** | 17 | 39932164 | T | C | intergenic | ORMDL3(dist=4563),LRRC3C(dist=9310) | AP-ALRI |
| **rs1808675** | 19 | 14203317 | A | G | intronic | ADGRL1 | AP-ALRI |
| **rs1811472** | 1 | 159672559 | G | C | intergenic | APCS(dist=83694),CRP(dist=39730) | AP-ATIN |
| **rs12093699** | 1 | 159678198 | G | A | intergenic | APCS(dist=89333),CRP(dist=34091) | AP-ATIN |
| **rs77013776** | 1 | 159682278 | A | C | intergenic | APCS(dist=93413),CRP(dist=30011) | AP-ATIN |
| **rs2592887** | 1 | 159683149 | C | T | intergenic | APCS(dist=94284),CRP(dist=29140) | AP-ATIN |
| **rs1470515** | 1 | 159683809 | C | T | intergenic | APCS(dist=94944),CRP(dist=28480) | AP-ATIN |
| **rs7415609** | 1 | 159685007 | T | C | intergenic | APCS(dist=96142),CRP(dist=27282) | AP-ATIN |
| **rs2592902** | 1 | 159685936 | G | T | intergenic | APCS(dist=97071),CRP(dist=26353) | AP-ATIN |
| **rs12749227** | 1 | 159689319 | C | T | intergenic | APCS(dist=100454),CRP(dist=22970) | AP-ATIN |
| **rs12754915** | 1 | 159691079 | T | C | intergenic | APCS(dist=102214),CRP(dist=21210) | AP-ATIN |
| **rs2808624** | 1 | 159696131 | C | G | intergenic | APCS(dist=107266),CRP(dist=16158) | AP-ATIN |
| **rs2794519** | 1 | 159698724 | T | C | intergenic | APCS(dist=109859),CRP(dist=13565) | AP-ATIN |
| **rs11265257** | 1 | 159699194 | C | T | intergenic | APCS(dist=110329),CRP(dist=13095) | AP-ATIN |
| **rs12741825** | 1 | 159700355 | C | T | intergenic | APCS(dist=111490),CRP(dist=11934) | AP-ATIN |
| **rs12755606** | 1 | 159700546 | C | G | intergenic | APCS(dist=111681),CRP(dist=11743) | AP-ATIN |
| **rs12754745** | 1 | 159700693 | G | A | intergenic | APCS(dist=111828),CRP(dist=11596) | AP-ATIN |
| **rs876537** | 1 | 159705143 | C | T | intergenic | APCS(dist=116278),CRP(dist=7146) | AP-ATIN |
| **rs2808628** | 1 | 159706221 | G | A | intergenic | APCS(dist=117356),CRP(dist=6068) | AP-ATIN |
| **rs2808629** | 1 | 159707006 | G | A | intergenic | APCS(dist=118141),CRP(dist=5283) | AP-ATIN |
| **rs2794520** | 1 | 159709026 | C | T | intergenic | APCS(dist=120161),CRP(dist=3263) | AP-ATIN |
| **rs1205** | 1 | 159712443 | C | T | UTR3 | CRP(NM_001382703:c.*1082A>G,NM_001329057:c.*374A>G,NM_001329058:c.*148A>G,NM_000567:c.*1082A>G) | AP-ATIN |
| **rs1130864** | 1 | 159713301 | G | A | UTR3 | CRP(NM_001382703:c.*224T>C,NM_000567:c.*224T>C) | AP-ATIN |
| **rs1800947** | 1 | 159713648 | C | G | exonic | CRP | AP-ATIN |
| **rs1417938** | 1 | 159714396 | T | A | intronic | CRP | AP-ATIN |
| **rs3091244** | 1 | 159714875 | G | A | upstream | CRP(dist=286) | AP-ATIN |
| **rs3116636** | 1 | 159716693 | G | A | intergenic | CRP(dist=2104),DUSP23(dist=64269) | AP-ATIN |
| **rs3116635** | 1 | 159716703 | A | G | intergenic | CRP(dist=2114),DUSP23(dist=64259) | AP-ATIN |
| **rs2369251** | 1 | 159717456 | A | G | intergenic | CRP(dist=2867),DUSP23(dist=63506) | AP-ATIN |
| **rs3122012** | 1 | 159719533 | T | C | intergenic | CRP(dist=4944),DUSP23(dist=61429) | AP-ATIN |
| **rs2027471** | 1 | 159719598 | T | A | intergenic | CRP(dist=5009),DUSP23(dist=61364) | AP-ATIN |
| **rs2211320** | 1 | 159723815 | G | A | intergenic | CRP(dist=9226),DUSP23(dist=57147) | AP-ATIN |
| **rs7551731** | 1 | 159724989 | T | C | intergenic | CRP(dist=10400),DUSP23(dist=55973) | AP-ATIN |
| **rs3116653** | 1 | 159727120 | G | C | intergenic | CRP(dist=12531),DUSP23(dist=53842) | AP-ATIN |
| **rs3116651** | 1 | 159728695 | C | T | intergenic | CRP(dist=14106),DUSP23(dist=52267) | AP-ATIN |
| **rs7553007** | 1 | 159728759 | G | A | intergenic | CRP(dist=14170),DUSP23(dist=52203) | AP-ATIN |
| **rs4546916** | 1 | 159729459 | G | T | intergenic | CRP(dist=14870),DUSP23(dist=51503) | AP-ATIN |
| **rs4261114** | 1 | 159731199 | A | C | intergenic | CRP(dist=16610),DUSP23(dist=49763) | AP-ATIN |
| **rs3116655** | 1 | 159731554 | C | T | intergenic | CRP(dist=16965),DUSP23(dist=49408) | AP-ATIN |
| **rs12727021** | 1 | 159732697 | G | A | intergenic | CRP(dist=18108),DUSP23(dist=48265) | AP-ATIN |
| **rs4287174** | 1 | 159733652 | T | A | intergenic | CRP(dist=19063),DUSP23(dist=47310) | AP-ATIN |
| **rs4428887** | 1 | 159733672 | A | G | intergenic | CRP(dist=19083),DUSP23(dist=47290) | AP-ATIN |
| **rs12037186** | 1 | 159736440 | A | G | intergenic | CRP(dist=21851),DUSP23(dist=44522) | AP-ATIN |
| **rs12759988** | 1 | 159738961 | G | A | intergenic | CRP(dist=24372),DUSP23(dist=42001) | AP-ATIN |
| **rs12042360** | 1 | 159739035 | G | A | intergenic | CRP(dist=24446),DUSP23(dist=41927) | AP-ATIN |
| **rs12728402** | 1 | 159740097 | C | G | intergenic | CRP(dist=25508),DUSP23(dist=40865) | AP-ATIN |
| **rs12728740** | 1 | 159740303 | C | A | intergenic | CRP(dist=25714),DUSP23(dist=40659) | AP-ATIN |
| **rs35198997** | 1 | 159740578 | T | C | intergenic | CRP(dist=25989),DUSP23(dist=40384) | AP-ATIN |
| **rs11265263** | 1 | 159740727 | C | A | intergenic | CRP(dist=26138),DUSP23(dist=40235) | AP-ATIN |
| **rs10437340** | 1 | 159741019 | G | C | intergenic | CRP(dist=26430),DUSP23(dist=39943) | AP-ATIN |
| **rs6672775** | 1 | 159742057 | T | A | intergenic | CRP(dist=27468),DUSP23(dist=38905) | AP-ATIN |
| **rs12730243** | 1 | 159742105 | A | T | intergenic | CRP(dist=27516),DUSP23(dist=38857) | AP-ATIN |
| **rs11265264** | 1 | 159743052 | G | A | intergenic | CRP(dist=28463),DUSP23(dist=37910) | AP-ATIN |
| **rs12083620** | 1 | 159743435 | T | A | intergenic | CRP(dist=28846),DUSP23(dist=37527) | AP-ATIN |
| **rs74596724** | 1 | 159743672 | A | G | intergenic | CRP(dist=29083),DUSP23(dist=37290) | AP-ATIN |
| **rs12049404** | 1 | 159744054 | C | T | intergenic | CRP(dist=29465),DUSP23(dist=36908) | AP-ATIN |
| **rs12760041** | 1 | 159744245 | C | T | intergenic | CRP(dist=29656),DUSP23(dist=36717) | AP-ATIN |
| **rs11265266** | 1 | 159744935 | G | A | intergenic | CRP(dist=30346),DUSP23(dist=36027) | AP-ATIN |
| **rs11588887** | 1 | 159747372 | G | A | intergenic | CRP(dist=32783),DUSP23(dist=33590) | AP-ATIN |
| **rs12734907** | 1 | 159749804 | A | T | intergenic | CRP(dist=35215),DUSP23(dist=31158) | AP-ATIN |
| **rs11811420** | 1 | 159750082 | G | C | intergenic | CRP(dist=35493),DUSP23(dist=30880) | AP-ATIN |
| **rs4131568** | 1 | 159752266 | C | T | intergenic | CRP(dist=37677),DUSP23(dist=28696) | AP-ATIN |
| **rs12094103** | 1 | 159753829 | G | A | intergenic | CRP(dist=39240),DUSP23(dist=27133) | AP-ATIN |
| **rs4512645** | 1 | 159759257 | G | A | intergenic | CRP(dist=44668),DUSP23(dist=21705) | AP-ATIN |
| **rs12726900** | 1 | 159762733 | G | A | intergenic | CRP(dist=48144),DUSP23(dist=18229) | AP-ATIN |
| **rs9273367** | 6 | 32658661 | A | T | downstream | HLA-DQB1(dist=807) | AP-ATIN |
| **rs9273368** | 6 | 32658698 | G | A | downstream | HLA-DQB1(dist=770) | AP-ATIN |
| **rs76192346** | 5 | 147804339 | A | G | intergenic | JAKMIP2(dist=21611),SPINK1(dist=20243) | AP-Bacterial Infection, AP-Cystitis, AP-IID, AP-Sepsis, AP-SSI, AP-VH |
| **rs559363229** | 5 | 147805265 | C | T | intergenic | JAKMIP2(dist=22537),SPINK1(dist=19317) | AP-Bacterial Infection, AP-Cystitis, AP-IID, AP-Sepsis, AP-SSI, AP-VH |
| **rs145092089** | 5 | 147806670 | T | G | intergenic | JAKMIP2(dist=23942),SPINK1(dist=17912) | AP-Bacterial Infection, AP-Cystitis, AP-IID, AP-Sepsis, AP-SSI, AP-VH |
| **rs140226647** | 5 | 147807668 | T | C | intergenic | JAKMIP2(dist=24940),SPINK1(dist=16914) | AP-Bacterial Infection, AP-Cystitis, AP-IID, AP-Sepsis, AP-SSI, AP-VH |
| **rs146437551** | 5 | 147808460 | T | G | intergenic | JAKMIP2(dist=25732),SPINK1(dist=16122) | AP-Bacterial Infection, AP-Cystitis, AP-IID, AP-Sepsis, AP-SSI, AP-VH |
| **rs77658106** | 5 | 147809858 | A | T | intergenic | JAKMIP2(dist=27130),SPINK1(dist=14724) | AP-Bacterial Infection, AP-Cystitis, AP-IID, AP-Sepsis, AP-SSI, AP-VH |
| **rs17107287** | 5 | 147813391 | C | T | intergenic | JAKMIP2(dist=30663),SPINK1(dist=11191) | AP-Bacterial Infection, AP-Cystitis, AP-IID, AP-Sepsis, AP-SSI, AP-VH |
| **rs17107294** | 5 | 147814680 | T | G | intergenic | JAKMIP2(dist=31952),SPINK1(dist=9902) | AP-Bacterial Infection, AP-Cystitis, AP-IID, AP-Sepsis, AP-SSI, AP-VH |
| **rs192858015** | 5 | 147815040 | G | A | intergenic | JAKMIP2(dist=32312),SPINK1(dist=9542) | AP-Bacterial Infection, AP-Cystitis, AP-IID, AP-Sepsis, AP-SSI, AP-VH |
| **rs145959667** | 5 | 147817967 | G | A | intergenic | JAKMIP2(dist=35239),SPINK1(dist=6615) | AP-Bacterial Infection, AP-Cystitis, AP-IID, AP-Sepsis, AP-SSI, AP-VH |
| **rs17107296** | 5 | 147818851 | A | C | intergenic | JAKMIP2(dist=36123),SPINK1(dist=5731) | AP-Bacterial Infection, AP-Cystitis, AP-IID, AP-Sepsis, AP-SSI, AP-VH |
| **rs145479930** | 5 | 147819449 | A | C | intergenic | JAKMIP2(dist=36721),SPINK1(dist=5133) | AP-Bacterial Infection, AP-Cystitis, AP-IID, AP-Sepsis, AP-SSI, AP-VH |
| **rs149882377** | 5 | 147825312 | C | T | intronic | SPINK1 | AP-Bacterial Infection, AP-Cystitis, AP-IID, AP-Sepsis, AP-SSI, AP-VH |
| **rs17107315** | 5 | 147828115 | T | C | exonic | SPINK1 | AP-Bacterial Infection, AP-Cystitis, AP-IID, AP-Sepsis, AP-SSI, AP-VH |
| **rs17107316** | 5 | 147829331 | T | C | intronic | SPINK1 | AP-Bacterial Infection, AP-Cystitis, AP-IID, AP-Sepsis, AP-SSI, AP-VH |
| **rs17107318** | 5 | 147829667 | A | G | intronic | SPINK1 | AP-Bacterial Infection, AP-Cystitis, AP-IID, AP-Sepsis, AP-SSI, AP-VH |
| **rs148276928** | 5 | 147833667 | T | C | intronic | SPINK1 | AP-Bacterial Infection, AP-Cystitis, AP-IID, AP-Sepsis, AP-SSI, AP-VH |
| **rs142703147** | 5 | 147835718 | C | A | intronic | SPINK1 | AP-Bacterial Infection, AP-Cystitis, AP-IID, AP-Sepsis, AP-SSI, AP-VH |
| **rs61905086** | 11 | 116744147 | T | C | intergenic | LINC02702(dist=85895),BUD13(dist=4023) | AP-Sepsis |
| **rs7853989** | 9 | 133256205 | G | C | exonic | ABO | AP-SSI |
| **rs2073823** | 9 | 133257129 | G | A | intronic | ABO | AP-SSI |
| **rs8176730** | 9 | 133257138 | T | C | intronic | ABO | AP-SSI |
| **rs8176725** | 9 | 133257230 | G | A | intronic | ABO | AP-SSI |
| **rs8176722** | 9 | 133257367 | C | A | intronic | ABO | AP-SSI |

**Table S4.** Pleiotropic Gene Identification

| **Cross_trait** | **Gene** | **CONTENT** | **fastBAT** | **MAGMA** | **PWAS** | **sCCA** | **SMR** | **Fine mapping** | **Count** |
| --- | --- | --- | --- | --- | --- | --- | --- | --- | --- |
| **AP-ALRI** | ABO |  |  |  | √ |  |  |  | 1 |
| **AP-ALRI** | AL355390.1 |  |  | √ |  |  |  |  | 1 |
| **AP-ALRI** | CTRB1 |  |  |  | √ |  |  |  | 1 |
| **AP-ALRI** | CTRB2 |  |  |  | √ |  |  |  | 1 |
| **AP-ALRI** | ERBB2 |  |  |  |  | √ |  |  | 1 |
| **AP-ALRI** | HAP1 |  |  | √ |  |  |  |  | 1 |
| **AP-ALRI** | KRT15 |  |  | √ |  |  |  |  | 1 |
| **AP-ALRI** | KRT19 |  |  | √ |  |  |  |  | 1 |
| **AP-ALRI** | KRT9 |  |  | √ |  |  |  |  | 1 |
| **AP-ALRI** | TBC1D3K |  | √ |  |  |  |  |  | 1 |
| **AP-ALRI** | TBC1D3D |  | √ |  |  |  |  |  | 1 |
| **AP-ALRI** | TBC1D3 |  | √ |  |  |  |  |  | 1 |
| **AP-ALRI** | TBC1D3C |  | √ |  |  |  |  |  | 1 |
| **AP-ALRI** | TBC1D3H |  | √ |  |  |  |  |  | 1 |
| **AP-ALRI** | YWHAEP7 |  | √ |  |  |  |  |  | 1 |
| **AP-ATIN** | ABO |  |  |  | √ |  |  |  | 1 |
| **AP-ATIN** | CTRB1 |  |  |  | √ |  |  |  | 1 |
| **AP-ATIN** | LAMTOR2 |  | √ |  |  |  |  |  | 1 |
| **AP-ATIN** | LMNA |  | √ |  |  |  |  |  | 1 |
| **AP-ATIN** | LTA4H |  | √ |  |  |  |  |  | 1 |
| **AP-ATIN** | MEX3A |  | √ |  |  |  |  |  | 1 |
| **AP-ATIN** | RAB25 |  | √ |  |  |  |  |  | 1 |
| **AP-ATIN** | UBQLN4 |  | √ |  |  |  |  |  | 1 |
| **AP-Bacterial Infection** | ABO |  |  |  | √ |  |  |  | 1 |
| **AP-Bacterial Infection** | CRP |  |  |  | √ |  |  |  | 1 |
| **AP-Bacterial Infection** | CTRB1 |  |  |  | √ |  |  |  | 1 |
| **AP-Bacterial Infection** | CTRB2 |  |  |  | √ |  |  |  | 1 |
| **AP-Bacterial Infection** | KCNE1 | √ |  |  |  |  |  |  | 1 |
| **AP-Bacterial Infection** | PFKFB4 | √ |  |  |  |  |  |  | 1 |
| **AP-Cystitis** | ALDH2 |  |  |  |  | √ |  |  | 1 |
| **AP-Cystitis** | CTRB1 |  |  |  | √ |  |  |  | 1 |
| **AP-Cystitis** | CUX2 |  |  | √ |  |  |  |  | 1 |
| **AP-Cystitis** | FLOT1 |  | √ |  |  |  |  |  | 1 |
| **AP-Cystitis** | HNRNPA3P1 |  | √ |  |  |  |  |  | 1 |
| **AP-Cystitis** | HNRNPF | √ |  |  |  |  |  |  | 1 |
| **AP-Cystitis** | IER3 |  | √ |  |  |  |  |  | 1 |
| **AP-Cystitis** | LTA4H |  | √ |  |  |  |  |  | 1 |
| **AP-Cystitis** | MAPKAPK5-AS1 |  |  |  |  | √ |  |  | 1 |
| **AP-Cystitis** | MIR6861 |  | √ |  |  |  |  |  | 1 |
| **AP-Cystitis** | MRAS |  |  | √ |  |  |  |  | 1 |
| **AP-Cystitis** | NAA25 |  | √ |  |  |  |  |  | 1 |
| **AP-Cystitis** | PGRMC2 | √ |  |  |  |  |  |  | 1 |
| **AP-Cystitis** | PPP1CC | √ |  |  |  |  |  |  | 1 |
| **AP-Cystitis** | SKAP1 | √ |  |  |  |  |  |  | 1 |
| **AP-Cystitis** | TRAFD1 |  | √ |  |  |  |  |  | 1 |
| **AP-Cystitis** | TRIM27 |  |  | √ |  |  |  |  | 1 |
| **AP-Cystitis** | TUBB |  | √ |  |  |  |  |  | 1 |
| **AP-IID** | ABO |  |  |  | √ |  |  |  | 1 |
| **AP-IID** | CTRB1 |  |  |  | √ |  |  |  | 1 |
| **AP-IID** | CTRB2 |  |  |  | √ |  |  |  | 1 |
| **AP-SSI** | HECTD4 |  | √ |  |  | √ |  |  | 2 |
| **AP-SSI** | KLHDC8B |  | √ |  |  | √ |  |  | 2 |
| **AP-SSI** | NAA25 |  | √ |  |  | √ |  |  | 2 |
| **AP-SSI** | PTPN11 |  | √ |  |  | √ |  |  | 2 |
| **AP-SSI** | TRAFD1 |  | √ |  |  | √ |  |  | 2 |
| **AP-SSI** | ABO |  |  |  | √ |  |  | √ | 2 |
| **AP-SSI** | ALDH2 |  |  |  |  | √ |  |  | 1 |
| **AP-SSI** | ALG2 |  |  |  |  | √ |  |  | 1 |
| **AP-SSI** | AMT |  |  |  |  | √ |  |  | 1 |
| **AP-SSI** | APOC3 |  |  |  | √ |  |  |  | 1 |
| **AP-SSI** | ARID3B | √ |  |  |  |  |  |  | 1 |
| **AP-SSI** | ATXN2 |  |  |  |  | √ |  |  | 1 |
| **AP-SSI** | BRAP |  |  |  |  | √ |  |  | 1 |
| **AP-SSI** | ERG28 |  | √ |  |  |  |  |  | 1 |
| **AP-SSI** | C3orf84 |  | √ |  |  |  |  |  | 1 |
| **AP-SSI** | IHO1 |  | √ |  |  |  |  |  | 1 |
| **AP-SSI** | CELSR3 |  |  |  |  | √ |  |  | 1 |
| **AP-SSI** | DAG1 |  |  |  |  | √ |  |  | 1 |
| **AP-SSI** | ERP29 |  | √ |  |  |  |  |  | 1 |
| **AP-SSI** | GPX1 |  |  |  |  | √ |  |  | 1 |
| **AP-SSI** | MAPKAPK5-AS1 |  |  |  |  | √ |  |  | 1 |
| **AP-SSI** | MIR3657 |  | √ |  |  |  |  |  | 1 |
| **AP-SSI** | MIR6861 |  | √ |  |  |  |  |  | 1 |
| **AP-SSI** | NICN1 |  |  |  |  | √ |  |  | 1 |
| **AP-SSI** | OAS1 |  | √ |  |  |  |  |  | 1 |
| **AP-SSI** | PCNPP1 |  |  |  |  | √ |  |  | 1 |
| **AP-SSI** | PRKAR2A |  |  |  |  | √ |  |  | 1 |
| **AP-SSI** | QARS1 |  |  |  |  | √ |  |  | 1 |
| **AP-SSI** | RPH3A |  | √ |  |  |  |  |  | 1 |
| **AP-SSI** | RPL6 |  | √ |  |  |  |  |  | 1 |
| **AP-SSI** | TCTA |  |  |  |  | √ |  |  | 1 |
| **AP-SSI** | USP4 |  |  |  |  | √ |  |  | 1 |
| **AP-SSI** | ZPR1 |  |  |  |  | √ |  |  | 1 |
| **AP-Sepsis** | ABO |  |  |  | √ |  |  |  | 1 |
| **AP-Sepsis** | CTRB1 |  |  |  | √ |  |  |  | 1 |
| **AP-Sepsis** | CTRB2 |  |  |  | √ |  |  |  | 1 |
| **AP-VH** | CTRB2 |  |  |  | √ | √ | √ |  | 3 |
| **AP-VH** | ABO |  |  |  | √ |  |  |  | 1 |
| **AP-VH** | APOC3 |  |  |  | √ |  |  |  | 1 |
| **AP-VH** | BCAR1 |  |  |  |  | √ |  |  | 1 |
| **AP-VH** | CTRB1 |  |  |  | √ |  |  |  | 1 |
| **AP-VH** | PGRMC2 | √ |  |  |  |  |  |  | 1 |
| **AP-ATIN** | CRP |  |  |  |  |  |  | √ | 1 |
| **AP-Bacterial Infection** | SPINK1 |  |  |  |  |  |  | √ | 1 |
| **AP-Cystitis** | SPINK1 |  |  |  |  |  |  | √ | 1 |
| **AP-IID** | SPINK1 |  |  |  |  |  |  | √ | 1 |
| **AP-Sepsis** | SPINK1 |  |  |  |  |  |  | √ | 1 |
| **AP-SSI** | SPINK1 |  |  |  |  |  |  | √ | 1 |
| **AP-VH** | SPINK1 |  |  |  |  |  |  | √ | 1 |

**Table S5.** sCCA Analysis Result

| **ID** | **panel_sCCA1_summary** | **panel_sCCA2_summary** | **panel_sCCA3_summary** | **acatP** | **Gene** | **Trait** | **FDR_P** |
| --- | --- | --- | --- | --- | --- | --- | --- |
| **ENSG00000141736.13** | 6.8618E-07 | 1.6722E-04 | 8.5492E-07 | 1.1394E-06 | ERBB2 | ALRI | 0.0150 |
| **ENSG00000111275.12** | 6.4409E-07 | 3.8649E-07 | 4.8383E-07 | 4.8334E-07 | ALDH2 | Cystitis | 0.0064 |
| **ENSG00000234608.7** | 1.1013E-05 | 7.0721E-06 | 5.6603E-06 | 7.3373E-06 | MAPKAPK5-AS1 | Cystitis | 0.0482 |
| **ENSG00000008300.16** | 4.0093E-04 | 2.1795E-01 | 1.2436E-05 | 3.6184E-05 | CELSR3 | SSI | 0.0317 |
| **ENSG00000089234.15** | 3.8057E-06 | 5.0628E-03 | 1.3929E-03 | 1.1377E-05 | BRAP | SSI | 0.0271 |
| **ENSG00000109917.10** | 1.9512E-05 | 2.4663E-01 | 7.9229E-03 | 5.8388E-05 | ZPR1 | SSI | 0.0404 |
| **ENSG00000111275.12** | 1.9259E-06 | 2.3463E-06 | 1.9235E-06 | 2.0473E-06 | ALDH2 | SSI | 0.0269 |
| **ENSG00000111300.9** | 3.6189E-04 | 4.0202E-04 | 8.6342E-06 | 2.4779E-05 | NAA25 | SSI | 0.0271 |
| **ENSG00000114302.15** | 5.3174E-05 | 4.1605E-03 | 2.5580E-05 | 5.1600E-05 | PRKAR2A | SSI | 0.0399 |
| **ENSG00000114316.12** | 1.2254E-05 | 5.6496E-03 | 5.4788E-05 | 2.9990E-05 | USP4 | SSI | 0.0281 |
| **ENSG00000119523.9** | 3.8750E-05 | 1.9749E-02 |  | 7.7349E-05 | ALG2 | SSI | 0.0484 |
| **ENSG00000135148.11** | 1.9103E-05 | 1.1754E-02 | 8.9994E-03 | 5.7094E-05 | TRAFD1 | SSI | 0.0404 |
| **ENSG00000145020.15** | 1.8146E-05 | 1.7686E-05 | 1.7968E-05 | 1.7931E-05 | AMT | SSI | 0.0271 |
| **ENSG00000145022.4** | 9.9093E-06 | 9.5158E-03 | 4.5447E-05 | 2.4385E-05 | TCTA | SSI | 0.0271 |
| **ENSG00000145029.13** | 7.7609E-06 | 6.3462E-06 | 7.0089E-06 | 6.9913E-06 | NICN1 | SSI | 0.0271 |
| **ENSG00000172053.17** | 4.4286E-05 |  | 5.9539E-06 | 1.0497E-05 | QARS1 | SSI | 0.0271 |
| **ENSG00000173064.12** | 2.5021E-05 | 1.4383E-03 | 3.7724E-03 | 7.3302E-05 | HECTD4 | SSI | 0.0482 |
| **ENSG00000173402.11** | 2.5578E-01 | 2.5578E-01 | 1.5026E-05 | 4.5073E-05 | DAG1 | SSI | 0.0370 |
| **ENSG00000179295.17** | 1.8138E-01 | 7.8842E-06 |  | 1.5768E-05 | PTPN11 | SSI | 0.0271 |
| **ENSG00000185909.14** | 1.1827E-05 | 1.6065E-05 | 1.7122E-05 | 1.4620E-05 | KLHDC8B | SSI | 0.0271 |
| **ENSG00000204842.14** | 1.5511E-02 | 1.4748E-05 | 1.4663E-05 | 2.2048E-05 | ATXN2 | SSI | 0.0271 |
| **ENSG00000233276.3** | 8.4548E-03 | 8.0945E-06 | 2.0398E-02 | 2.4251E-05 | GPX1 | SSI | 0.0271 |
| **ENSG00000234608.7** | 7.0831E-06 | 6.9391E-06 | 6.8262E-06 | 6.9479E-06 | MAPKAPK5-AS1 | SSI | 0.0271 |
| **ENSG00000258359.1** | 1.6034E-05 | 1.6448E-02 | 2.4204E-05 | 2.8917E-05 | PCNPP1 | SSI | 0.0281 |
| **ENSG00000050820.16** | 5.6688E-03 | 2.1371E-07 | 2.3131E-02 | 6.4109E-07 | BCAR1 | VH | 0.0042 |
| **ENSG00000168928.12** | 3.1594E-07 | 1.0018E-02 |  | 6.3186E-07 | CTRB2 | VH | 0.0042 |

**Table S6.** fastBAT-GCTA Analysis Result

| **Gene** | **Chr** | **Start** | **End** | **No.SNPs** | **Chisq(Obs)** | **Pvalue** | **TopSNP.Pvalue** | **TopSNP** | **Trait** | **FDR_P** |
| --- | --- | --- | --- | --- | --- | --- | --- | --- | --- | --- |
| **LOC101060351** | 17 | 37924471 | 37935371 | 41 | 285.598 | 3.42993E-07 | 4.7026E-07 | rs2952157 | ALRI | 0.0042 |
| **LOC101060376** | 17 | 37978154 | 37989062 | 34 | 177.97 | 3.81785E-06 | 3.77175E-06 | rs12939566 | ALRI | 0.0110 |
| **LOC101060376** | 17 | 38004018 | 38014908 | 36 | 188.53 | 4.9104E-06 | 3.77175E-06 | rs12939566 | ALRI | 0.0110 |
| **LOC101060389** | 17 | 37978154 | 37989048 | 34 | 177.97 | 3.81785E-06 | 3.77175E-06 | rs12939566 | ALRI | 0.0110 |
| **LOC101060389** | 17 | 38004032 | 38014908 | 36 | 188.53 | 4.9104E-06 | 3.77175E-06 | rs12939566 | ALRI | 0.0110 |
| **LOC101060389** | 17 | 38057692 | 38068594 | 51 | 280.386 | 2.69616E-05 | 3.77175E-06 | rs12939566 | ALRI | 0.0473 |
| **LOC102724862** | 17 | 37978154 | 37989048 | 34 | 177.97 | 3.81785E-06 | 3.77175E-06 | rs12939566 | ALRI | 0.0110 |
| **LOC102724862** | 17 | 38004032 | 38014908 | 36 | 188.53 | 4.9104E-06 | 3.77175E-06 | rs12939566 | ALRI | 0.0110 |
| **TBC1D3** | 17 | 37924459 | 37935554 | 41 | 285.598 | 3.42993E-07 | 4.7026E-07 | rs2952157 | ALRI | 0.0042 |
| **TBC1D3** | 17 | 38057509 | 38068606 | 51 | 280.386 | 2.69616E-05 | 3.77175E-06 | rs12939566 | ALRI | 0.0473 |
| **TBC1D3C** | 17 | 38003992 | 38138868 | 118 | 576.697 | 0.00001686 | 3.77175E-06 | rs12939566 | ALRI | 0.0345 |
| **TBC1D3H** | 17 | 37978098 | 37989062 | 34 | 177.97 | 3.81785E-06 | 3.77175E-06 | rs12939566 | ALRI | 0.0110 |
| **TBC1D3H** | 17 | 38004018 | 38014964 | 36 | 188.53 | 4.9104E-06 | 3.77175E-06 | rs12939566 | ALRI | 0.0110 |
| **YWHAEP7** | 17 | 37842948 | 37884743 | 59 | 338.276 | 1.42055E-06 | 2.90122E-07 | rs2934967 | ALRI | 0.0110 |
| **LAMTOR2** | 1 | 156054725 | 156058510 | 58 | 310.944 | 2.16553E-06 | 4.59987E-07 | rs3814314 | ATIN | 0.0106 |
| **LMNA** | 1 | 156082545 | 156140089 | 91 | 387.415 | 1.93923E-06 | 4.59987E-07 | rs3814314 | ATIN | 0.0106 |
| **LTA4H** | 12 | 96000752 | 96043520 | 208 | 634.23 | 9.68964E-06 | 1.29635E-05 | rs4762572 | ATIN | 0.0397 |
| **MEX3A** | 1 | 156072012 | 156081998 | 65 | 334.327 | 1.92835E-06 | 4.59987E-07 | rs3814314 | ATIN | 0.0106 |
| **RAB25** | 1 | 156061174 | 156070504 | 68 | 349.263 | 1.51331E-06 | 4.59987E-07 | rs3814314 | ATIN | 0.0106 |
| **UBQLN4** | 1 | 156035300 | 156053725 | 64 | 340.452 | 1.31244E-06 | 4.59987E-07 | rs3814314 | ATIN | 0.0106 |
| **FLOT1** | 6 | 30727733 | 30742676 | 136 | 578.154 | 9.25723E-06 | 3.42109E-07 | rs3130660 | Cystitis | 0.0325 |
| **HNRNPA3P1** | 10 | 43787411 | 43790417 | 105 | 383.331 | 5.90872E-06 | 6.03993E-06 | rs57479451 | Cystitis | 0.0325 |
| **IER3** | 6 | 30743198 | 30744550 | 128 | 561.687 | 8.81774E-06 | 3.42109E-07 | rs3130660 | Cystitis | 0.0325 |
| **LTA4H** | 12 | 96000752 | 96043520 | 208 | 641.405 | 8.00969E-06 | 2.04897E-06 | rs10777712 | Cystitis | 0.0325 |
| **MIR6861** | 12 | 112163257 | 112163321 | 29 | 152.723 | 7.05042E-06 | 1.45648E-06 | rs610303 | Cystitis | 0.0325 |
| **NAA25** | 12 | 112026688 | 112108831 | 44 | 247.093 | 6.84319E-06 | 1.45648E-06 | rs610303 | Cystitis | 0.0325 |
| **TRAFD1** | 12 | 112125544 | 112153604 | 31 | 170.712 | 7.51769E-06 | 1.45648E-06 | rs610303 | Cystitis | 0.0325 |
| **TUBB** | 6 | 30720379 | 30725418 | 132 | 551.119 | 1.19568E-05 | 3.42109E-07 | rs3130660 | Cystitis | 0.0367 |
| **C3orf84** | 3 | 49177635 | 49191858 | 33 | 150.285 | 1.47169E-05 | 8.24702E-06 | rs9834003 | SSI | 0.0386 |
| **C14orf1** | 14 | 75650889 | 75661195 | 71 | 287.777 | 1.64588E-05 | 5.22447E-05 | rs7494514 | SSI | 0.0386 |
| **CCDC36** | 3 | 49198427 | 49258104 | 43 | 180.263 | 1.15795E-05 | 8.24702E-06 | rs9834003 | SSI | 0.0386 |
| **ERP29** | 12 | 112013347 | 112023220 | 31 | 160.501 | 0.000021971 | 0.000019934 | rs9300319 | SSI | 0.0386 |
| **HECTD4** | 12 | 112160187 | 112382092 | 57 | 267.277 | 3.28303E-06 | 3.66957E-06 | rs632650 | SSI | 0.0269 |
| **KLHDC8B** | 3 | 49171584 | 49176486 | 36 | 164.664 | 1.75229E-05 | 8.24702E-06 | rs9834003 | SSI | 0.0386 |
| **MIR3657** | 12 | 112037598 | 112037715 | 28 | 155.553 | 2.12474E-05 | 1.78646E-05 | rs10774632 | SSI | 0.0386 |
| **MIR6861** | 12 | 112163257 | 112163321 | 29 | 171.01 | 1.48178E-06 | 3.66957E-06 | rs632650 | SSI | 0.0199 |
| **NAA25** | 12 | 112026688 | 112108831 | 44 | 249.901 | 5.92393E-06 | 3.66957E-06 | rs632650 | SSI | 0.0364 |
| **OAS1** | 12 | 112906933 | 112919907 | 30 | 140.632 | 2.09846E-05 | 5.84111E-06 | rs11066320 | SSI | 0.0386 |
| **PTPN11** | 12 | 112418731 | 112509913 | 37 | 162.468 | 1.24477E-05 | 4.2973E-06 | rs17696736 | SSI | 0.0386 |
| **RPH3A** | 12 | 112791743 | 112898879 | 42 | 172.631 | 1.51587E-05 | 5.84111E-06 | rs11066320 | SSI | 0.0386 |
| **RPL6** | 12 | 112405189 | 112409639 | 20 | 107.296 | 2.02532E-05 | 0.000009604 | rs76300790 | SSI | 0.0386 |
| **TRAFD1** | 12 | 112125544 | 112153604 | 31 | 191.399 | 1.61814E-06 | 3.66957E-06 | rs632650 | SSI | 0.0199 |

**Table S7.** MAGMA Analysis Result

| **ID** | **Gene** | **CHR** | **START** | **STOP** | **NSNPS** | **NPARAM** | **Z** | **P** | **Trait** | **FDR_P** |
| --- | --- | --- | --- | --- | --- | --- | --- | --- | --- | --- |
| **ENSG00000171345** | KRT19 | 17 | 39679869 | 39684560 | 9 | 2 | 4.2345 | 1.15E-05 | ALRI | 0.0434 |
| **ENSG00000171346** | KRT15 | 17 | 39669995 | 39678781 | 41 | 2 | 4.3094 | 8.18E-06 | ALRI | 0.0434 |
| **ENSG00000171403** | KRT9 | 17 | 39722096 | 39728310 | 4 | 1 | 4.7134 | 1.22E-06 | ALRI | 0.0225 |
| **ENSG00000173805** | HAP1 | 17 | 39873994 | 39890896 | 42 | 3 | 4.2283 | 1.18E-05 | ALRI | 0.0434 |
| **ENSG00000177596** | AL355390.1 | 13 | 74987094 | 74993252 | 12 | 3 | 4.3234 | 7.68E-06 | ALRI | 0.0434 |
| **ENSG00000111249** | CUX2 | 12 | 111471828 | 111788358 | 237 | 12 | 4.4347 | 4.61E-06 | Cystitis | 0.0496 |
| **ENSG00000158186** | MRAS | 3 | 138066539 | 138124375 | 123 | 8 | 4.3179 | 7.88E-06 | Cystitis | 0.0496 |
| **ENSG00000204713** | TRIM27 | 6 | 28870779 | 28891766 | 87 | 13 | 4.3127 | 8.07E-06 | Cystitis | 0.0496 |

**Table S8.** CONTENT Analysis Result

| **PANEL** | **Gene** | **CHR** | **Start** | **End** | **BEST.GWAS.ID** | **TWAS.Z** | **TWAS.P** | **Trait** | **FDR_P** |
| --- | --- | --- | --- | --- | --- | --- | --- | --- | --- |
| **plasmacytoid Dendritic cell** | KCNE1 | 21 | 34446688 | 34512210 | rs13052531 | 4.556484225 | 5.20169E-06 | Bacterial_infection | 0.031124508 |
| **CD8-positive T cell** | PFKFB4 | 3 | 48517684 | 48562015 | rs9834003 | 4.486162352 | 7.25175E-06 | Bacterial_infection | 0.031124508 |
| **CD8-positive T cell** | HNRNPF | 10 | 43385617 | 43409166 | rs2505510 | -5.064767712 | 4.08899E-07 | Cystitis | 0.001754993 |
| **CD8-positive T cell** | PGRMC2 | 4 | 128269237 | 128288829 | rs6851935 | 5.094205172 | 3.50207E-07 | Cystitis | 0.001754993 |
| **Non-Classical Monocyte cell** | PPP1CC | 12 | 110719680 | 110742939 | rs12369009 | -4.356919625 | 1.31906E-05 | Cystitis | 0.031213119 |
| **CD4-positive T cell** | SKAP1 | 17 | 48133442 | 48430275 | rs858677 | 4.335471682 | 1.45448E-05 | Cystitis | 0.031213119 |
| **Non-Classical Monocyte cell** | ARID3B | 15 | 74541177 | 74598131 | rs11072518 | 4.578741741 | 4.67781E-06 | SSI | 0.040154349 |
| **CD8-positive T cell** | PGRMC2 | 4 | 128269237 | 128288829 | rs9991981 | 4.555125397 | 5.23543E-06 | VH | 0.044940963 |

**Table S9.** PWAS Analysis Result

| **PANEL** | **Gene** | **CHR** | **P0** | **P1** | **HSQ** | **TWAS.Z** | **TWAS.P** | **Trait** | **FDR_P** |
| --- | --- | --- | --- | --- | --- | --- | --- | --- | --- |
| **Plasma_Protein** | ABO | 9 | 133233278 | 133276024 | 0.181754 | 4.830275662 | 1.36344E-06 | ALRI | 0.001742478 |
| **Plasma_Protein** | CTRB2 | 16 | 75204103 | 75207161 | 0.387819 | -4.071204803 | 4.67706E-05 | ALRI | 0.019924278 |
| **Plasma_Protein** | CTRB1 | 16 | 75218988 | 75226338 | 0.16513 | 4.346369879 | 1.38409E-05 | ALRI | 0.008844341 |
| **Plasma_Protein** | ABO | 9 | 133233278 | 133276024 | 0.181754 | 4.842310172 | 1.28338E-06 | ATIN | 0.001640162 |
| **Plasma_Protein** | CTRB1 | 16 | 75218988 | 75226338 | 0.16513 | 3.95490745 | 7.65643E-05 | ATIN | 0.048924578 |
| **Plasma_Protein** | CRP | 1 | 159712289 | 159714589 | 0.060345 | 4.382311974 | 1.17426E-05 | Bacterial | 0.005119361 |
| **Plasma_Protein** | ABO | 9 | 133233278 | 133276024 | 0.181754 | 4.934955101 | 8.01692E-07 | Bacterial | 0.001024562 |
| **Plasma_Protein** | CTRB2 | 16 | 75204103 | 75207161 | 0.387819 | -4.147564999 | 3.3603E-05 | Bacterial | 0.010736161 |
| **Plasma_Protein** | CTRB1 | 16 | 75218988 | 75226338 | 0.16513 | 4.377274134 | 1.20173E-05 | Bacterial | 0.005119361 |
| **Plasma_Protein** | CTRB1 | 16 | 75218988 | 75226338 | 0.16513 | 4.113295775 | 3.9005E-05 | Cystitis | 0.049848404 |
| **Plasma_Protein** | ABO | 9 | 133233278 | 133276024 | 0.181754 | 4.377198535 | 1.20214E-05 | IID | 0.007681704 |
| **Plasma_Protein** | CTRB2 | 16 | 75204103 | 75207161 | 0.387819 | -3.91822017 | 8.92052E-05 | IID | 0.038001419 |
| **Plasma_Protein** | CTRB1 | 16 | 75218988 | 75226338 | 0.16513 | 4.409355626 | 1.03679E-05 | IID | 0.007681704 |
| **Plasma_Protein** | ABO | 9 | 133233278 | 133276024 | 0.181754 | 4.599857221 | 4.22781E-06 | Sepsis | 0.002701568 |
| **Plasma_Protein** | CTRB2 | 16 | 75204103 | 75207161 | 0.387819 | -4.362000787 | 1.28878E-05 | Sepsis | 0.00549022 |
| **Plasma_Protein** | CTRB1 | 16 | 75218988 | 75226338 | 0.16513 | 4.610073974 | 4.02526E-06 | Sepsis | 0.002701568 |
| **Plasma_Protein** | ABO | 9 | 133233278 | 133276024 | 0.181754 | 5.337707235 | 9.41293E-08 | SSI | 0.000120297 |
| **Plasma_Protein** | APOC3 | 11 | 116829706 | 116833072 | 0.013834 | 4.162430134 | 3.14879E-05 | SSI | 0.020120736 |
| **Plasma_Protein** | ABO | 9 | 133233278 | 133276024 | 0.181754 | 5.032820191 | 4.83316E-07 | VH | 0.000308839 |
| **Plasma_Protein** | APOC3 | 11 | 116829706 | 116833072 | 0.013834 | 4.09795634 | 4.16814E-05 | VH | 0.013317203 |
| **Plasma_Protein** | CTRB2 | 16 | 75204103 | 75207161 | 0.387819 | -5.052210706 | 4.36726E-07 | VH | 0.000308839 |
| **Plasma_Protein** | CTRB1 | 16 | 75218988 | 75226338 | 0.16513 | 4.832328012 | 1.34946E-06 | VH | 0.000574869 |


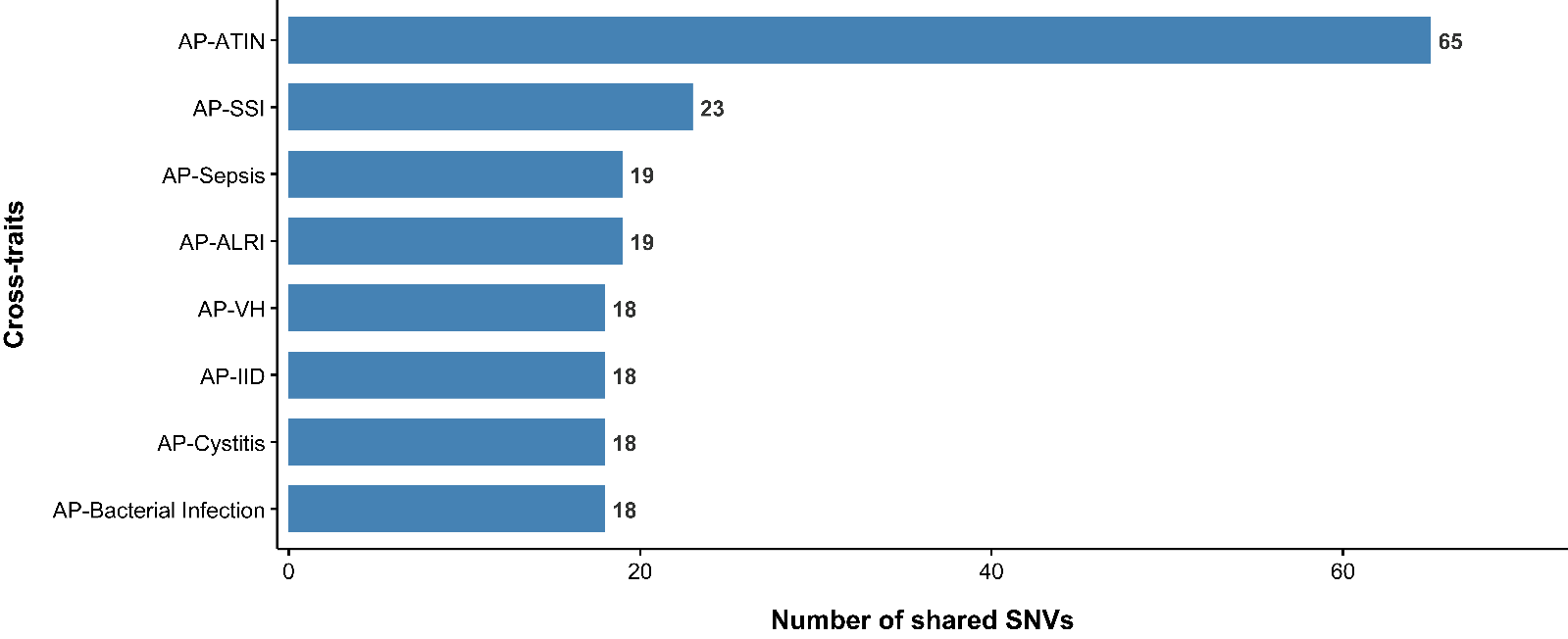


**Figure S1.** Number of Shared Variants in Each Trait-pair.


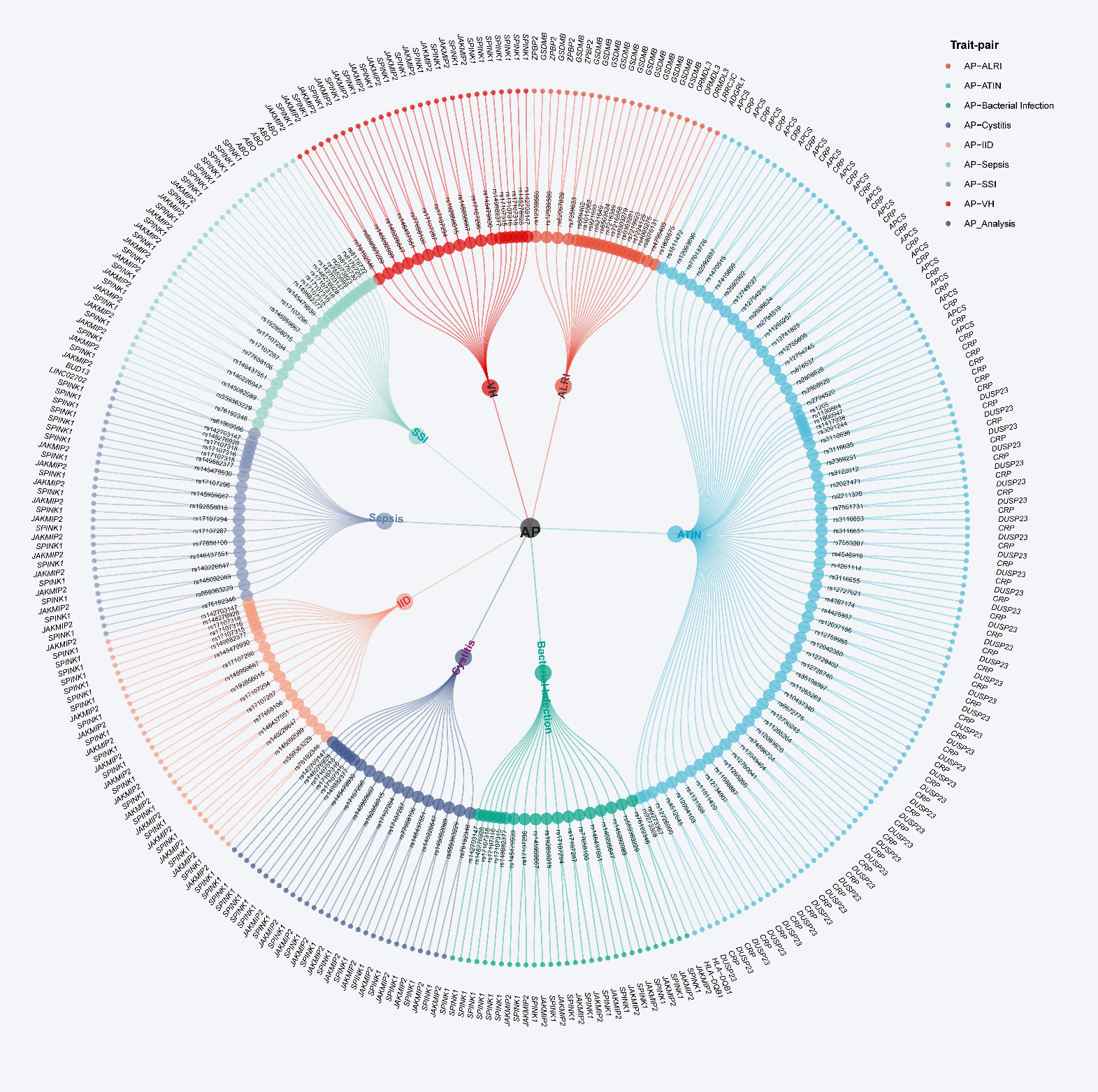


**Figure S2.** Identification of Pleiotropic Loci Using Cross-Trait Meta-Analysis


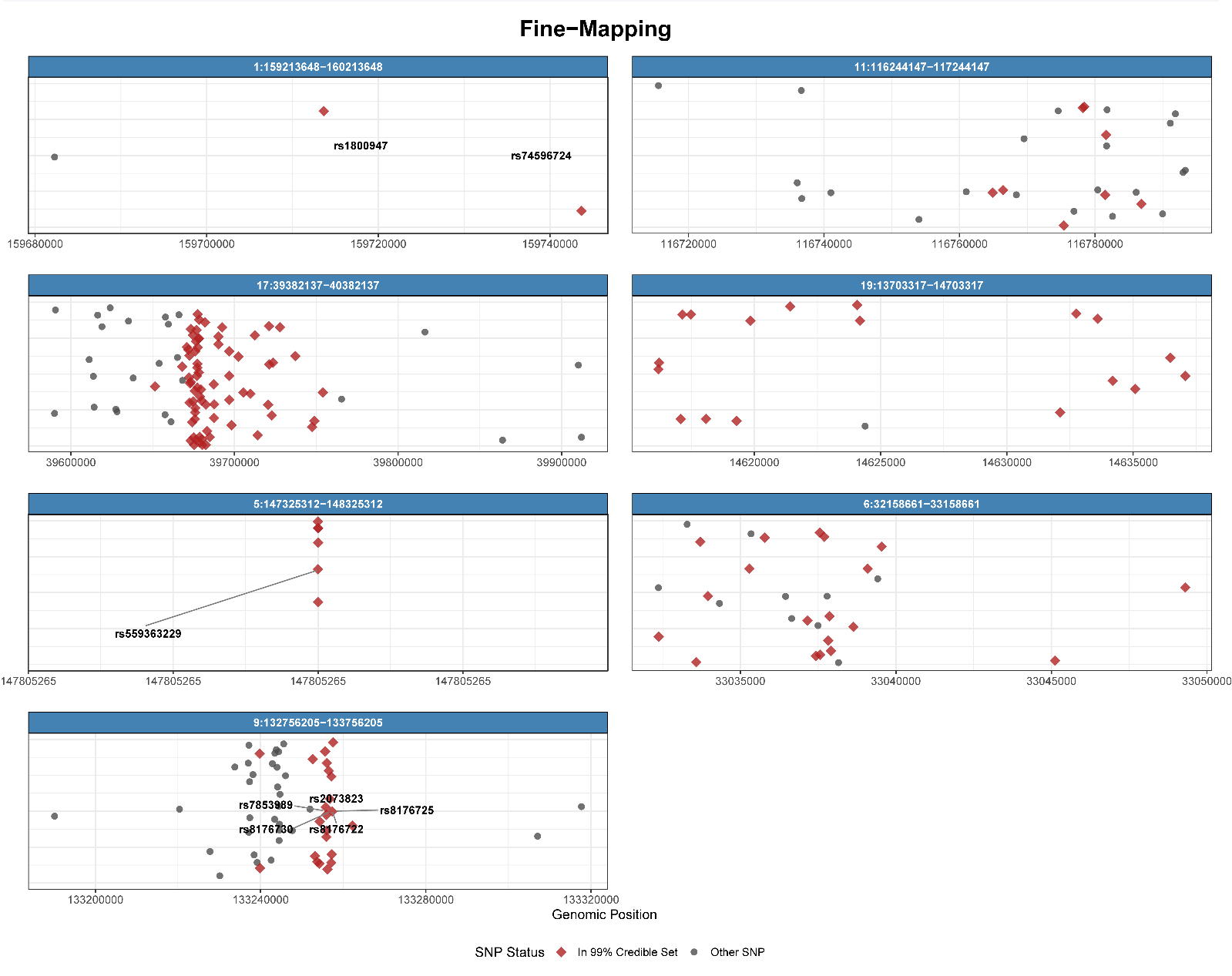


**Figure S3.** Bayesian Fine-Mapping of Pleiotropic Loci to Prioritize Causal Variants.


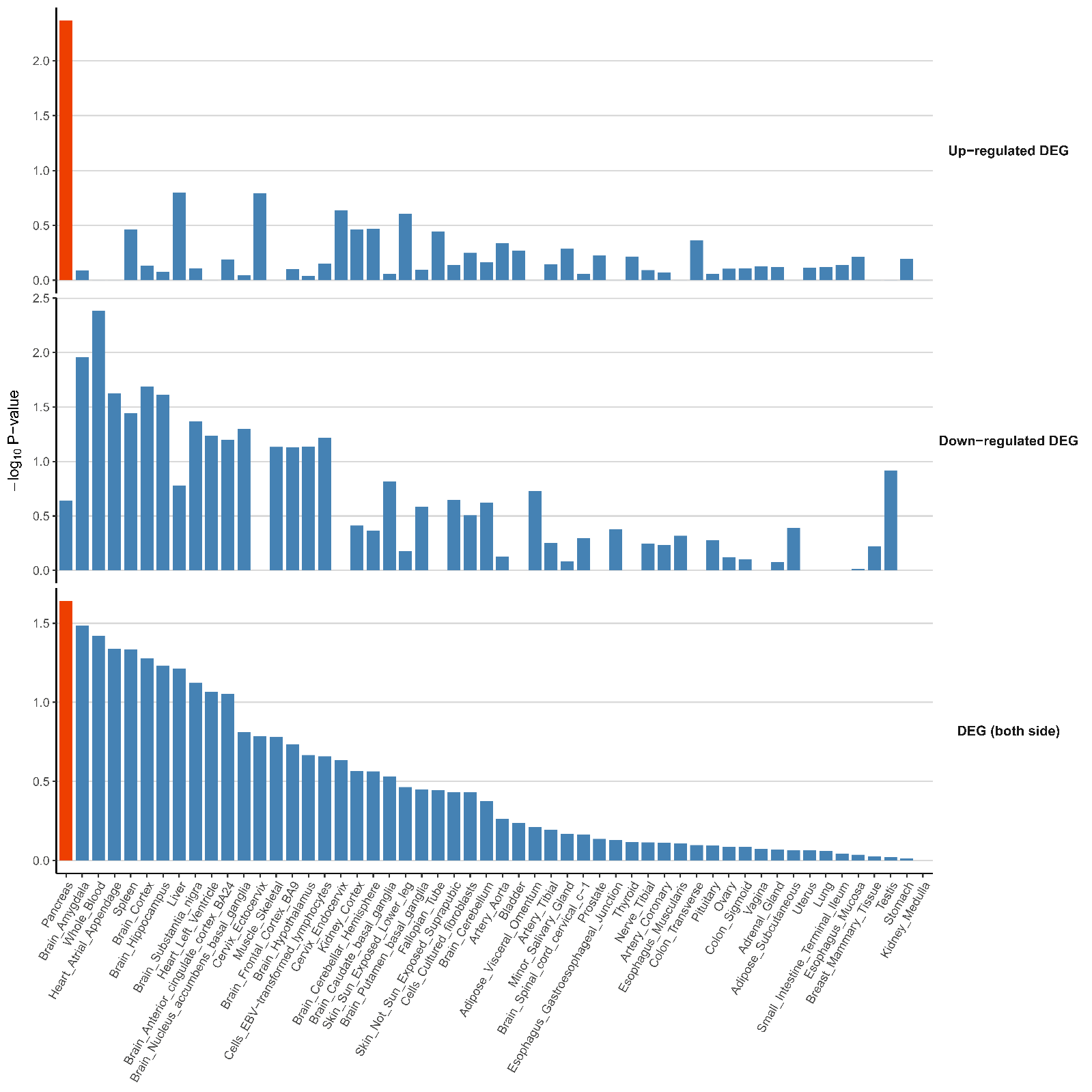


**Figure S4.** Differential Expressed Gene Analysis.


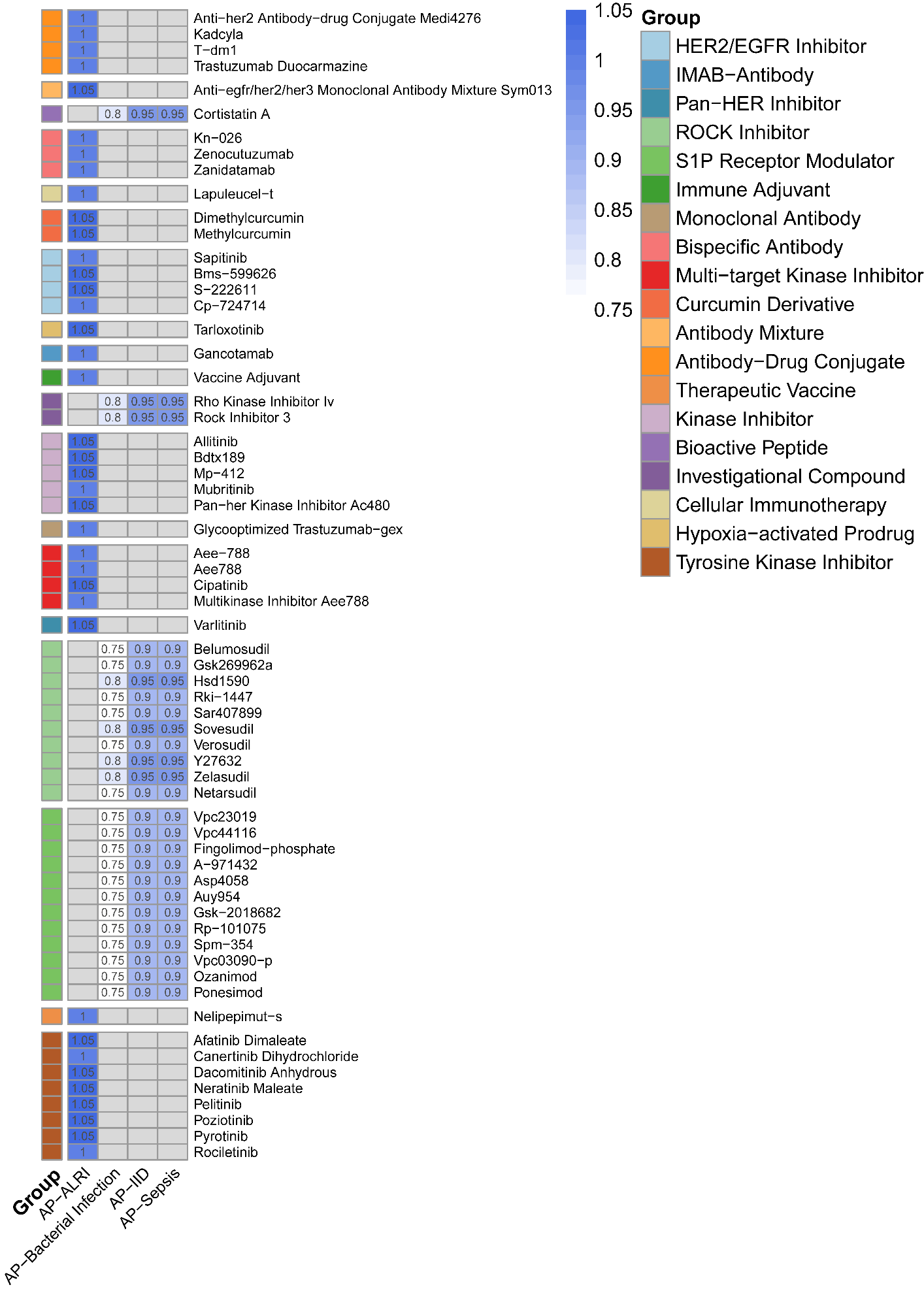


**Figure S5.** Pathway Pairing Score for Systematic Drug Repurposing. Heatmap of pairing scores for candidate drugs across eight AP-infection trait pairs. Higher scores indicate stronger alignment between a drug's pharmacological pathways and the disease's pathological pathways.
